# Multiple Evolutionary Origins of Ubiquitous Cu^2+^ and Zn^2+^ Binding in the S100 Protein Family

**DOI:** 10.1371/journal.pone.0164740

**Published:** 2016-10-20

**Authors:** Lucas C. Wheeler, Micah T. Donor, James S. Prell, Michael J. Harms

**Affiliations:** 1 Department of Chemistry and Biochemistry, University of Oregon, Eugene, Oregon, 97403, United States of America; 2 Institute for Molecular Biology, University of Oregon, Eugene, Oregon, 97403, United States of America; Russian Academy of Medical Sciences, RUSSIAN FEDERATION

## Abstract

The S100 proteins are a large family of signaling proteins that play critical roles in biology and disease. Many S100 proteins bind Zn^2+^, Cu^2+^, and/or Mn^2+^ as part of their biological functions; however, the evolutionary origins of binding remain obscure. One key question is whether divalent transition metal binding is ancestral, or instead arose independently on multiple lineages. To tackle this question, we combined phylogenetics with biophysical characterization of modern S100 proteins. We demonstrate an earlier origin for established S100 subfamilies than previously believed, and reveal that transition metal binding is widely distributed across the tree. Using isothermal titration calorimetry, we found that Cu^2+^ and Zn^2+^ binding are common features of the family: the full breadth of human S100 paralogs—as well as two early-branching S100 proteins found in the tunicate *Oikopleura dioica*—bind these metals with μM affinity and stoichiometries ranging from 1:1 to 3:1 (metal:protein). While binding is consistent across the tree, structural responses to binding are quite variable. Further, mutational analysis and structural modeling revealed that transition metal binding occurs at different sites in different S100 proteins. This is consistent with multiple origins of transition metal binding over the evolution of this protein family. Our work reveals an evolutionary pattern in which the overall phenotype of binding is a constant feature of S100 proteins, even while the site and mechanism of binding is evolutionarily labile.

## Introduction

The S100 protein family is an important group of calcium binding proteins found in vertebrates [1,2]. Humans possess 27 family members that play diverse functional roles in inflammation [3–5], cell proliferation [6–8], and innate immunity [9–11]. S100 proteins are particularly prominent in inflammatory diseases and cancers, where they are used both as clinical markers and drug targets [12–21]. S100 proteins are found only in chordates and are highly diverged from other calcium binding proteins [2,12].

**Fig 1:**
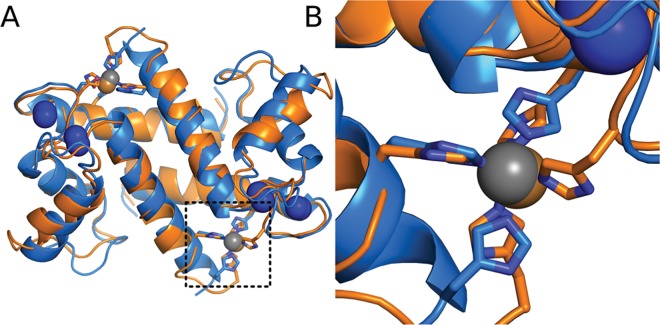
Transition metal binding occurs at a common site in diverse S100 proteins. Overlay of the crystal structures of S100B (orange, PDB 3CZT) and S100A12 (blue, PDB 1ODB) bound to Ca^2+^ and transition metals. Ions are shown as colored spheres: Ca^2+^ (blue), Zn^2+^ (gray) and Cu^2+^ (copper). Residues ligating the transition metals are are shown as sticks. Boxed region is shown in detail in panel B.

Most S100 proteins share a common homodimeric structure in which ~10 kDa monomers come together to form a compact α-helical fold (Fig 1A). Each monomer binds two Ca^2+^ ions in conserved calcium binding motifs, inducing a conformational change that exposes a hydrophobic surface [22–24]. This surface can then interact with and modulate the activity of downstream target proteins [25,26].

In addition to Ca^2+^, many S100 proteins interact with divalent transition metals such as Zn^2+^, Cu^2+^, or Mn^2+^ as part of their biological functions [27,28]. Such functions include metal transport [29], modulation of signaling [30], and antimicrobial activity [10]. Their transition metal binding constants tend to be ~μM, consistent with their roles in metal transport and metal-dependent signaling [31,32]. Despite the importance played by these metals, transition metal binding has not been studied systematically across the family [27,28]. While one key transition metal site—at the dimer interface—has been studied extensively (Fig 1B), the transition metal binding capacity of many S100 proteins remains unknown. For many others, there are conflicting reports about the binding affinities, sites, and stoichiometries for binding to divalent transition metals [27,28].

Evolutionary history provides a powerful lens through which to understand this metal binding diversity and its accompanying functional diversity. Understanding when a feature evolved in the family, and thus which homologs might share the feature, helps translate observations for one family member into predictions about other family members. One key question is whether transition metal binding is a shared ancestral feature, or whether it has been acquired independently on multiple lineages. Although all five crystal structures of S100 proteins bound to transition metals have similar binding sites (Fig 1B), experimental evidence suggests that other S100s bind to divalent transition metals at a different site than the one identified crystallographically [33,34], consistent with at least one more acquisition of transition metal binding.

A well-supported phylogeny of the S100 protein family would allow observations of transition metal binding to be mapped as evolutionary characters, thereby allowing inferences about the evolutionary history of the character. Several phylogenies have been published [2,12,35–37], however, these trees are not fully consonant with one another, making interpretation difficult. Previous analyses were limited by the number of S100 sequences available, particularly from early-branching vertebrate species. Further, all but one [36] relied on distance-based phylogenetic methods. Increased taxonomic sampling, combined with more advanced phylogenetic methods, will provide a much clearer picture of S100 evolution.

We therefore set out to understand the evolution of transition metal binding in this family through a combination of phylogenetic analysis and biochemical characterization of select human paralogs. Further, to establish the ancient features of the family, we performed the first-ever biochemical characterization of two early-diverging S100 proteins from the tunicate *Oikopleura dioica*. Our work sheds light on the evolutionary process that gave the diversity of modern S100 proteins, as well as revealing the broad-brush evolution of the transition-metal binding phenotype of this important protein family.

## Results

### The S100 family arose in the ancestor of *Olfactores*

Our first goal was to establish the taxonomic distribution of the S100 family. We began with an iterative BLAST approach. We used the full set of 27 human S100 family members (S1 Table) as a starting point for PSI-BLAST against the NCBI non-redundant protein database. In addition to identifying thousands of S100 sequences, this protocol picked up non-S100 calcium binding proteins such as calmodulin and troponin, indicating that we had saturated S100 proteins in the database. We filtered our hits by reverse BLAST. All S100 hits were within vertebrates, with the exception of four hits from the tunicate *Oikopleura dioica*. To further support the taxonomic distribution of the S100s, we then used BLAST to search directly in the genomes and transcriptomes from representative tunicates, cephalochordates, hemichordates, and echinoderms. Only a transcriptome from the tunicate *Molgula tectiform* yielded a further S100 hit. We also queried the HMMER database, but found no new S100 family members. The presence of S100 proteins in tunicates and vertebrates (Olfactores), but not other chordates, suggests that the first S100 arose in the last common ancestor of tunicates and vertebrates, ~700 million years ago [38]. These results are consistent with previous studies that noted the relative youth of the S100 family [2,12,36,37].

### Model-based phylogenetic approaches reveal well-supported clades

We next constructed a phylogenetic tree, using sequences drawn from across Olfactores. Phylogenetic analyses of this family are challenging as it is large and diverse. For example, the average sequence identity of the 27 human family members is 29.5%, with the most divergent pair (A3 and A14) only 13.2% identical. Further, the small size of these proteins (~100 amino acids) means they have few evolutionary characters and, thus, relatively weak phylogenetic signal. Finally, many S100 paralogs exhibit highly specific tissue distributions, meaning that transcriptomes can provide very incomplete pictures of the S100 complement of a given organism.

To construct a tree despite these difficulties, we assembled a high-quality dataset of 564 sequences, from 52 species, through targeted searches of key genome/transcriptome/proteome databases (S2 Table, S1 Spreadsheet). In an effort to bracket the class-level evolutionary origin of each S100 ortholog—despite incomplete sequence data and possible differential loss along each lineage—we included multiple species within each class: two Tunicata (one Ascidiacea, one Appendicularia), two Agnathan (jawless fishes), seven Chondrichthyans (cartilaginous fishes), eight Actinopterygii (ray-finned fishes), three Sarcopterygii (lobe-finned fishes), seven Amphibians, fourteen Sauropsids (birds and reptiles), and seven Mammals (two monotremes, two therians, and three eutherians). We generated a 133 character alignment from these sequences (S1 Fig and S2 Fig, S1 Alignment) and used this for model-based phylogenetics.

**Fig 2:**
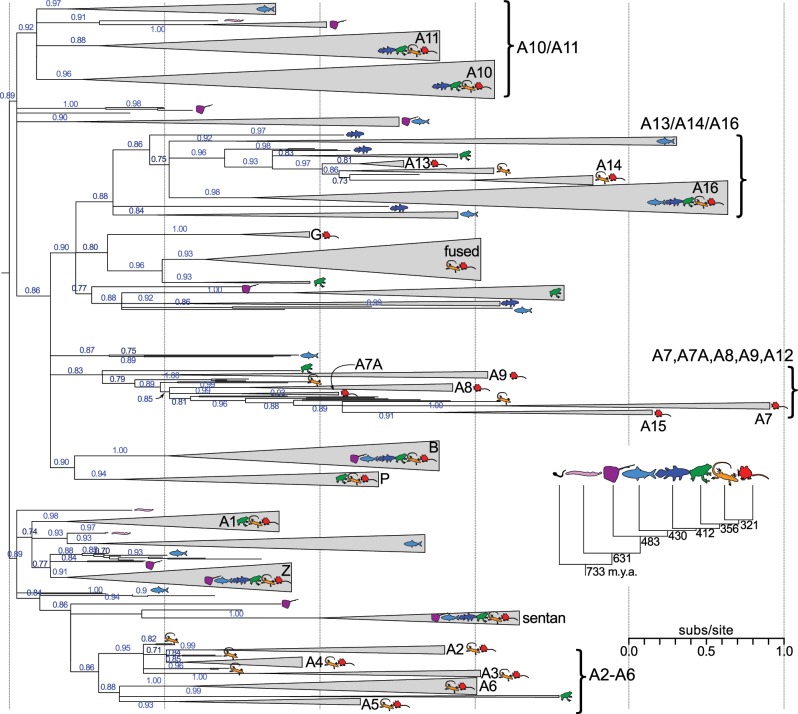
Model-based phylogenetics reveal several S100 subfamilies. Maximum likelihood phylogeny of 564 S100 proteins drawn from 52 *Olfactores* species. Wedges are collapsed clades of shared orthologs, with wedge height denoting number of included taxa and wedge length denoting longest branch length with the clade. Support values are SH-supports, derived from an approximate likelihood ratio test. Rooting is arbitrary, but roughly balances the distribution of jawless fishes across the ancestral node. Icons indicate taxonomic classes represented within each clade: tunicates (black), jawless fishes (pink), cartilaginous fishes (purple), ray-finned fishes (light blue), lobe-finned fishes (blue), amphibans (green), birds/reptiles (yellow), and mammals (red). Inset shows estimated divergence times for each taxonomic class in millions of years before present.

We used both maximum likelihood (ML) and Bayesian approaches to construct phylogenetic trees for the family (Fig 2, S1 Tree and S2 Tree, S3 Fig). Both approaches resolved well-supported clades containing each of the human seed paralogs. This allowed us to assign the orthology, relative to the human proteins, for 500 of the 564 sequences in our data set (S1 Spreadsheet). In addition, the ML and Bayesian approaches revealed a set of consonant clades: A2/A3/A4; A5/A6; the calgranulins (A7/A8/A9/A12); A13/A14; and the so-called “fused” family (cornulin/ trichohyalin/repetin/hornerin/filaggrin) (Fig 2 and S3 Fig). In the Bayesian consensus tree, no further relationships could be resolved. Several other clades were resolved in the ML tree (Fig 2); A2/A3/A4 groups with A4/A5; A10 with A11; and A13/A14 groups with A16. In both trees, the sum of the branch lengths was extremely long, reflecting the high diversity of the family.

We were particularly interested in placing the tunicate S100 proteins on the tree. If we could assign the orthology of these proteins, we could potentially identify the most ancient S100 orthlog(s). Unfortunately, the placement of these sequences on the tree was neither evolutionarily reasonable nor stable between phylogenetic runs. For example, a single tunicate protein might end up on a long branch within a clade of mammalian proteins in one analysis, and then in an entirely different location in another. We thus excluded the tunicate proteins from the final phylogenetic analysis.

Uncertainty in the deepest branching pattern precluded rooting of the tree. We attempted to root the phylogeny by three methods; however, none proved successful. The first method was to include non-S100 calcium-binding proteins identified in our BLAST searches (sentan, calcineurins, troponins, and calmodulins) as an outgroup. With the exception of sentan, these non-S100 proteins grouped together; however, the branch leading to the clade was too long to allow robust placement relative to the S100 proteins—minor changes to the alignment and/or tree-building protocol would radically change their relationship to the rest of the tree. We also attempted to use the tunicate proteins, but as they could not be placed, this was ineffective. Finally, we attempted to minimize the number of duplications and losses across the tree; however, the lack of resolution of the deepest nodes also made identifying the precise origin (and thus gain/loss) of each paralog problematic.

### Synteny and taxonomic distribution further support relationships among S100 proteins

**Fig 3:**
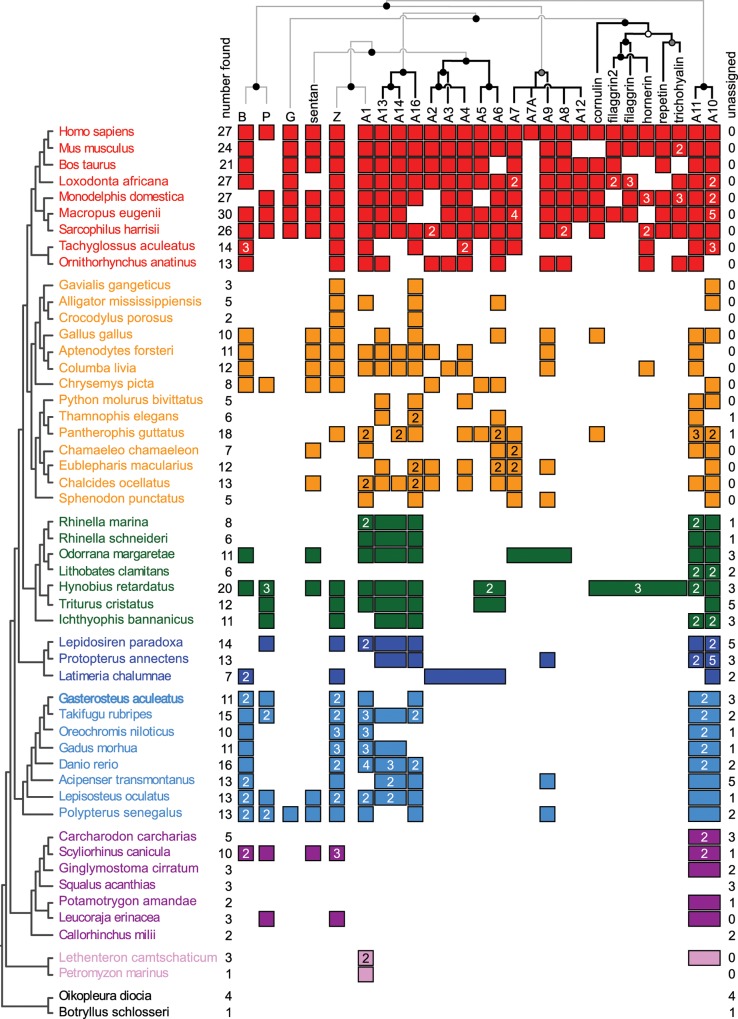
Model-based phylogeny, synteny, and taxonomic distribution provide a consonant picture of S100 evolution. The human S100 orthologs are shown across the top, in the order they occur in the human genome. B, P, G, and Z occur on different chromosomes; A1-A10 are in a contiguous region of chromosome I. Sentan, an evolutionary relative, is also on a different chromosome. Species are shown on the left, organized by taxonomy. Color indicates taxonomic class, as in Fig 2. Squares denote the presence of an ortholog to the human gene for each species; a number in the box indicates the number of co-orthologous genes found in that species (if more than one); squares fused into a rectangle indicate a gene found in an earlier branching lineage that subsequently duplicated somewhere along the lineage leading to *Homo sapiens*. Total number of genes found for each species are shown on the left. The number of genes that were not orthologous to human genes (or could not be classified) are shown on right. Top tree shows the maximum-likelihood phylogeny of the family mapped onto the S100 genes found in the human genome. Circles denote SH support ≥ 0.85 (black); ≥ 0.75 (gray), < 0.75 (white). Branches supported by both the ML phylogeny and synteny are shown in black; branches supported by only the ML tree are shown in gray.

Because model-based phylogenetic methods provided relatively weak support for relationships within in the family, we used the taxonomic distribution of orthologs and synteny to further support the relationships we observed in the model-based approaches. Fig 3 shows distribution of observed orthologs to human genes across the species included in our analysis. (Species phylogenies taken from [39–47]). We mapped these orthologs onto the arrangement of these genes in the human genome (top). Four S100 genes (G, B, P, and Z) are scattered on different chromosomes, while twenty-two S100 genes (A1 through A10) form a contiguous block on a single chromosome. This tight linkage group has been noted previously [12,37,48], and arose at least as early as the bony vertebrates [37].

There is strong correlation between the S100 subfamilies identified in model-based phylogenetics and the distribution of the genes across human chromosome I. Proteins with shared evolutionary relationships form blocks across this region, suggesting local expansion by gene duplication. The ML relationship between orthologs are shown above the plot in Fig 3. The clades identified in our model-based phylogenetics form individually contiguous blocks: A13-A16, A2-A6, A7-A12, S100-fused, and A11-A10. This consonance between the phylogenetic signal and genomic arrangement supports the shared ancestry of these subfamilies.

The species distribution of these orthologs then provides insight into the diversification of the family. For example, A10, A11, or their common ancestor (A10/A11) are found in all vertebrates, demonstrating that this protein arose no later than the last common ancestor of vertebrates. Because some genes may have been missed within each species—either through lineage-specific loss or incomplete genomic/transcriptomic coverage—this is a lower bound on the age of the gene. After its origin, A10/A11 then diversified in later lineages. In the bony fishes, A10 expanded, as reflected in the increased numbers of genes co-orthologous to A10/A11. A10/A11 gave rise to the tetrapod paralogs A10 and A11 via tandem gene duplication in the ancestor of the lobe-finned fishes.

Another ancient S100 by this analysis is A1, which, intriguingly, brackets the other end of the contiguous S100 genome region mammals and some fishes [37]. The simplest interpretation of this pattern would be that the A1 or A10/A11 gene was the earliest gene in this syntenic block, and that the remaining family arose by serial expansion from that starting point.

Other ancient S100 orthologs are B, P, and Z. Our tree provides some evidence that A1 and Z share a common ancestor, and that B and P share a common ancestor. Intriguingly, these four ancient proteins are scattered throughout vertebrate genomes, rather than being a part of the expanded gene region containing A1-A10. This suggests that the last common ancestor of jawed vertebrates had a collection of four to five S100 proteins, but that only the region containing A1-A10 then continued to expand with the radiation of the vertebrates. Sentan—a close evolutionary relative to the S100 family that does not possess the diagnostic pseudo EF-hand of true family members—also arose in the early vertebrates. Given the ambiguity of the deepest branching of the tree, it is unclear whether it is an out group or, instead, a duplication of an established S100 paralog.

The gene block containing A1-A10 expanded by what appears to be a set of local gene duplication events. A13/A14 and A16 likely arose next, at least by the ancestor of bony vetebrates. Like A10/A11, these genes were duplicated through the whole genome duplications of teleost fishes, giving rise to multiple S100 genes that are co-orthogolous to the human genes in bony fishes. The tetrapod paralogs A13 and A14 did not arise until the amniotes, when they formed via duplication from A13/A14. The next phase of expansion was local duplication that led to the ancestors of A2-A6, A7-A12, and the S100-fused proteins in early tetrapods. These founding genes then expanded across the tetrapods, with several duplicates preserved in Sauropsids. The final mammalian complement was achieved by several more duplications. The A7-A12 and S100-fused clades—which are directly adjacent in mammalian chromosomes—continue to rapidly expand by duplication.

### Transition metal binding is nearly universal across the family

**Fig 4:**
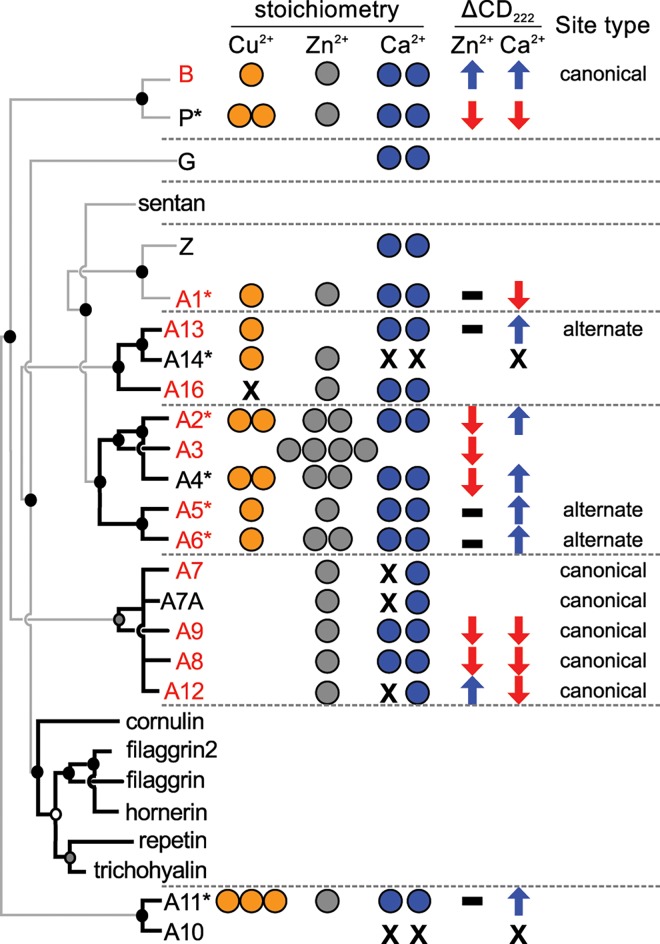
Transition metal binding is widely distributed across the S100 family. The human S100 paralogs are shown on the left, organized as on the top of Fig 3. Asterisks indicate S100 proteins investigated in the current study; red color indicates a protein for which transition metal binding has been noted in the literature previously. Biochemical properties of the human paralogs are shown as columns. Circles denote stoichiometry of binding for Cu^2+^ (orange), Zn^2+^ (gray), and Ca^2+^ (blue). “X” indicates that the protein does not bind the metal; empty space is unmeasured. Arrows indicate the change in far-UV CD signal with the indicated metal: no change (black), increase (blue), and decrease (red). The transition metal binding site is indicated as “canonical” (B-like) or “alternate” (some other site).

With the phylogenetic tree in hand, we next set out to determine the distribution of transition metal binding across the tree. Previously reported transition metal binding is scattered across the tree (Fig 4, red proteins) [27,28]. If this feature were ancestral, we predicted that transition metal binding would be present across the majority of the tree. To test this hypothesis we used isothermal titration calorimetry (ITC) to measure the ability of human S100 proteins to bind to Zn^2+^ and Cu^2+^—the two most prevalent transition metals encountered biologically—under approximately physiological conditions (125 mM ionic strength, pH 7.4, 25°C). We chose proteins that would maximize the sampling across clades. Some of the proteins we selected have been reported to bind transition metals, albeit with variable stoichiometry [34,49]. The other paralogs have, to our knowledge, yet to be characterized.

We found that Zn^2+^ and Cu^2+^ binding was universally distributed across the tree: every single S100 protein we characterized bound to Zn^2+^ and/or Cu^2+^ with low micromolar affinity (Fig 4 and S4 Fig, S3 Table)[10,34,50–55]. With one exception, stoichiometry ranged from 1:1 to 3:1 (metal:monomer). These binding affinities and stoichiometries are similar to previously measured transition metal binding affinities for S100 proteins [28,50,54,56]. Buffer-specific enthalpies ranged from -5.4 to 6.1 kcal/mol; the majority of the enthalpies were negative. All of the proteins tested bound to both Zn^2+^ and Cu^2+^, with the exception of A1 which did not bind Cu^2+^ under our experimental conditions. The Zn^2+^ binding isotherm for A6 and the Cu^2+^ binding isotherms for A2 and A4 were not well fit by standard binding models (as is often observed for metal binding studies by ITC: [57]), however, from the curves we could gain insight into their stoichiometry. The A6/Zn^2+^ and A4/Cu^2+^ curves exhibited two phases, consistent with two binding sites. The A2/Cu^2+^ curve was quite broad, consistent with >2 metals binding per monomer. Representative binding isotherms for Zn^2+^ and Cu^2+^ to a variety of S100 proteins—including the three problematic curves—are shown in S4 Fig. All measured thermodynamic parameters are reported in S3 Table.

We next asked if the structural response to these metals, like the binding constant, was consistent across the tree. We measured Zn^2+^-induced changes in secondary structure by comparing the far-UV circular dichroism (CD) spectra of these proteins with EDTA versus saturating Zn^2+^ (S5 Fig). We found the response was variable across the family (Fig 4) [34,50,54,58–65]. For some proteins, Zn^2+^ induced a decrease in CD signal (P, A2 and A4); in others, it had no effect (A1, A11, A5 and A6). We also observed Zn^2+^-induced protein precipitation in the case of A14, which was rapidly reversible by the addition of excess EDTA. We also asked whether the structural response to Zn^2+^ exhibited by these proteins correlated with the response to their canonical agonist Ca^2+^. We found that they were largely uncorrelated (Fig 4 and S5 Fig). For example, P has decreased CD signal with both Zn^2+^ and Ca^2+^, while A2 shows decreased signal with Zn^2+^ and increased signal with Ca^2+^.

When placed onto the phylogenetic tree, a few patterns in these responses emerge (Fig 4). Phylogenetically close members of the family appear to display similar structural responses to Zn^2+^ binding. For example, the closely related A2 and A4 proteins show qualitatively similar decreases in CD signal in the presence of Zn^2+^ relative to the apo form. Likewise, the far-UV CD signal of direct sister proteins A5 and A6 is insensitive to Zn^2+^. This said, such patterns are not universal. For example, B and P are directly sister but have opposite structural responses to Zn^2+^. Further, family members exhibit all possible combinations of increased and decreased CD signal with the addition of Ca^2+^ and Zn^2+^, revealing the variability of this trait over evolutionary time.

### Early-diverging tunicate S100s bind transition metals

Given that all human paralogs we characterized were capable of binding transition metals, we predicted that this was a conserved, early feature of the protein family. To test this prediction, we turned to two tunicate homologs, which represent some of the earliest-diverging S100 proteins. We selected two *Oikopleura dioica* proteins—tunA (tunicate A, CBY12809.1) and tunB (tunicate B, CBY30360.1)—for characterization. Although the orthology of these proteins is unclear, the proteins sample the breadth of tunicate S100 diversity, exhibiting only 26.2% identity. We expressed and purified these proteins, and then characterized their metal binding features.

**Fig 5:**
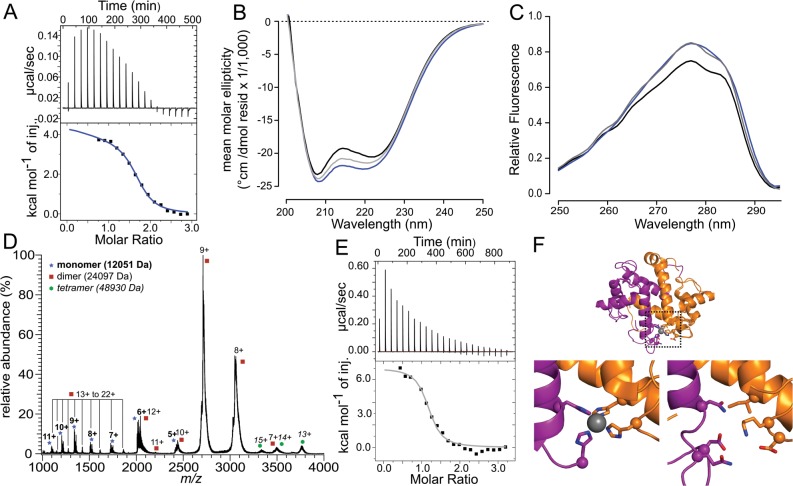
Early-branching tunicate S100 binds transition metals at a non-canonical site. Colors indicate the metal present during experiment: Zn^2+^ is gray, Ca^2+^ is blue. A) Ca^2+^ binding to tunB by ITC. Top panel shows power traces for injections; bottom curve shows integrated heats and model fit to extract thermodynamic parameters. B) Far-UV circular dichroism spectra of the apo protein (black), Ca^2+^ bound protein (blue), or Zn^2+^ bound protein (gray). C) Intrinsic fluorescence spectra, with samples colored as in panel B. D) Mass spectrum of tunB. Notes above each peak indicate molecular weight and corresponding oligomeric state. E) Zn^2+^ binding to tunB by ITC, with subpanels as in A. E) Homology model of tunB overlaid on crystal structure of human S100B (PBD: 3ZCT). Ligating residues are shown as sticks, with C_α_ atoms shown as spheres. A and B chains of the dimer are shown in orange and purple, respectively. Zn^2+^ ion is shown as gray sphere. Top panel shows overlay, with box highlighting the zoomed-up regions shown at right. Bottom left panel shows S100B structure with Zn^2+^ chelation. Bottom left panel shows tunB homology model, highlighting residues that would have to chelate Zn^2+^.

Because these proteins have not been characterized previously, we first performed a baseline characterization to verify that they behave like other S100 proteins. We first measured Ca^2+^ binding. Like many other S100 proteins, both tunA and tunB bound Ca^2+^ with nanomolar to micromolar dissociation constants and 2:1 (per monomer) stoichiometry (Fig 5A and S6 Fig). Further, both proteins exhibited changes in secondary and/or tertiary structure—as measured by far-UV circular dichroism (CD) and intrinsic fluorescence—with the addition of saturating amounts of Ca^2+^ (Fig 5B and 5C and S6 Fig). All of the observed changes were strictly metal dependent and reversible upon the addition of EDTA. Metal-dependent changes in conformation, as reflected in these changes in spectroscopic signals, are a hallmark of S100 proteins [34,66–68].

We then assessed the ability of these proteins to form homodimers—a key feature of most S100 proteins—using native electrospray-ionization mass spectrometry (nanoESI) [69]. For tunB, we detected homodimers (Fig 5D). The narrow distribution of relatively low charge states observed in the nanoESI mass spectra for both the monomer and dimer ions indicate that the proteins are not denatured under these conditions and undergo little unfolding during the ionization process. The broad mass spectral peaks observed are the result of adduction of residual sodium from solution that has survived buffer exchange. To see if the dimer peaks were the result of non-specific aggregation during the electrospray process, we measured dimerization at protein concentrations at which non-specific dimerization is not expected (< 1 μM, see methods). We found homodimers, even at 10 nM protein, consistent with a specific tunB dimer (S7 Fig). We also observed a small amount of homotetramer; however, the tetramer was not robust to dilution and is likely an artifact of the electrospray process (S7 Fig). For tunA, we detected homodimers; however, these were not robust to dilution, suggesting that dimerization is relatively weak for this protein (S6 Fig). We corroborated these observations for tunA and tunB using a sedimentation velocity experiment (S8 Fig). Under these conditions, we found that tunB was primarily a dimer. In contrast, tunA exhibited both monomer and dimer species, consistent with this protein forming a weaker dimer. Further work is required to determine the precise distribution of oligomeric species in solution for these proteins; however, these results are consistent with both proteins having the ability to form homodimers, like other S100 proteins [70].

**Fig 6:**
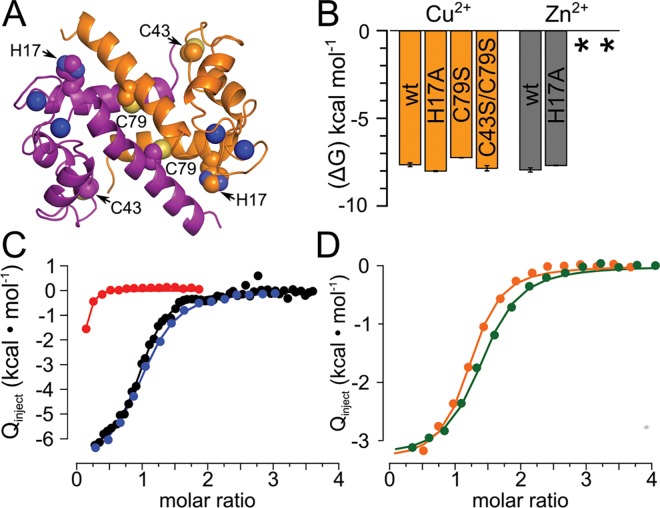
Human S100A5 does not bind transition metals at the same site as B and the calgranulins. A) Mutated residues mapped onto the NMR structure of Ca^2+^-bound human A5 (PDB: 2KAY). Dimer chains are colored purple and orange. H17, C43, C79 and Ca^2+^ ions are shown as spheres. The location of H17 corresponds to the transition metal site in calgranulins and B (Fig 1B); C43 and C79 are in different regions of S100A5. C) Binding free energies measured for Cu^2+^ (copper) and Zn^2+^ (gray) to human A5 and its mutants. Zn^2+^ binding constants could not be extracted for the C43S and C43S/C79S proteins (*). C) Integrated heats for ITC titration of Zn^2+^ onto A5 (black), A5/H17A (blue) and A5/C43S (red). D) Integrated heats for ITC titration of Cu^2+^ onto A5 in the absence (orange) or presence (green) of saturating (500 μM) Zn^2+^.

We next turned our attention to Zn^2+^ binding. By ITC, both tunA and tunB bound to Zn^2+^ with nM to μM affinity and stoichiometries of 2:1 (Fig 5E and S6 Fig). We attempted to verify these stoichiometries by ESI-MS; however, we were unable to disentangle specific from non-specific metal adduction in these samples. We then measured the changes in secondary and tertiary structure measured by far-UV CD and intrinsic tyrosine fluorescence. Although both proteins bound Zn^2+^ tightly, only tunB displayed a pronounced structural response, similar to that induced by Ca^2+^ binding (Figs 5B and 6C). The secondary structure of tunA was insensitive to Zn^2+^ binding although the protein displayed a moderate increase in intrinsic tyrosine fluorescence (S6 Fig).

### Transition metal binding occurs at independently evolved binding sites

The broad distribution of transition metal binding across human paralogs, along with the observed transition metal binding in the early-branching tunicate proteins, suggests that transition metal binding is an essentially universal property of this family. We next sought to understand to what extent transition metal binding across the family reflects a common binding site, or rather convergent acquisition of metal binding on multiple lineages. Transition metal binding to S100 proteins has been extensively characterized in B and the “calgranulin” clade (A7,A8,A9,A12,A15), where it occurs at the same site, using similar ligating residues (Fig 1B). B is an ancient protein, arising at least as early as the cartilaginous fishes (Fig 3). In contrast, the calgranulins arose ~80 million years later in the ancestor of amniotes (Fig 3). If the common site reflects shared ancestry, we would expect to observe the same site across a wide variety of descendants—possibly explaining the ubiquity of transition-metal binding across the tree.

We first investigated the clade containing A2,A3,A4,A5, and A6. All members of this clade possess a conserved histidine that, in B and the calgranulins, coordinates transition metals (Fig 6A). We chose to investigate human A5, as it binds to both Zn^2+^ and Cu^2+^ with 1:1 stoichiometry, and thus simplifies identification of the binding site. We mutated His17 to Ala in human A5 and measured metal binding of the mutant. Surprisingly the H17A mutation had only a small effect on Zn^2+^ binding (1.3 +/- 0.3 to 3.0 +/- 0.1 μM), suggesting it is not directly involved in the binding of Zn^2+^ in human A5. Additionally, this mutation did not compromise Cu^2+^ binding (Fig 6B). Previous reports suggested that Cys residues in the loop between helices 2 and 3, as well as those near the N and C-termini, could play a role in binding divalent transition metals in this clade [27,34,51]. We therefore mutated these residues to serine in A5 and measured binding of Zn^2+^ and Cu^2+^ to the mutants. Mutating the C-terminal Cys (C79S) had no effect on Cu^2+^ binding, but led to a drastic change in the Zn^2+^ binding curve (Fig 6C). The apparent stoichiometry of binding was drastically reduced (~0.1), which is consistent with only a small fraction of the protein being competent to bind Zn^2+^. Additionally, the enthalpy of binding is mostly ablated. These results clearly indicate that C79 is involved in Zn^2+^, but not Cu^2+^ binding. We attempted to ablate Cu^2+^ binding by also mutating the loop Cys residue (C43S), but found that this double Cys mutant (C43S/C79S) still left Cu^2+^ binding unaffected (Fig 6B). These results show that Zn^2+^ and Cu^2+^ not only bind outside the B/calgranulin site, but bind at different sites on the same protein. To confirm that these metals bind at different sites, we also measured binding of Cu^2+^ to Zn^2+^-saturated human A5 and found no evidence of competition between the two metals (Fig 6D). Finally, because mutating H17, C43, and C79 did not disrupt Cu^2+^ binding, we hypothesized that the metal might bind at one of the Ca^2+^ binding motifs. We therefore repeated the Cu^2+^ binding curve in the presence of saturating (2 mM) Ca^2+^. We observed extensive aggregation, however, which made interpretation of the ITC binding isotherm impossible. This suggests that previously-noted antagonism between Ca^2+^ and Cu^2+^ [54] may be an artifact of aggregation rather than true antagonism.

We next turned our attention to the tunicate protein tunB. This protein behaves like a conventional S100 protein, forming a homodimer, binding to Ca^2+^ and changing its structure in response to metals (Fig 5A–5E). Further, it binds to transition metals with a 2:1 stoichiometry. To determine if it could bind metals at the canonical transition metal binding site, we constructed a homology model for the protein and then inspected the residues that would form the S100B/calgranulin binding pocket. These are Asp, Gln, Asn, and Lys (Fig 5F). The lack of a His or Cys residue suggests this site is not capable of binding transition metals. Thus, transition metal binding in this early-branching ortholog almost certainly occurs at a different site.

## Discussion

Our work provides a high-level view of the evolution of the S100 protein family and the ability of its members to bind to divalent transition metals. Our work provides the best-resolved phylogeny yet determined for this family. All characterized human paralogs, as well as two early-branching tunicate S100 homologs, bind to transition metals with a physiologically relevant ~μM binding constant. On the other hand, different S100 proteins bind at different transition metal binding sites. Thus, the apparently “conserved” feature of transition metal binding actually reflects independent acquisition of metal binding on multiple lineages. Further, the structural changes induced by transition metal binding are variable, suggesting quite different mechanisms of binding and possible functional consequences for different family members.

### Multiple origins of transition metal binding

Our work, combined with previous publications, reveals at least four sites—and therefore four evolutionary origins—of transition metal binding in the S100 family: the B/calgranulin site (Fig 1B), A5's Cys-79 site (Fig 6), an N-terminal Cys in A2 [34], and a unique glutamate-rich site in human A13 [33]. The plasticity of this feature is likely because of the relative ease, biochemically, of creating transition metal binding sites [71–73]. A few amino acid substitutions can create a new site, while a few other substitutions ablate an existing site. This is similar to the evolutionary behavior of phosphorylation sites, which can shift rapidly over evolutionary time [74]. Additionally, some of the proteins may bind to transition metals in one of the Ca^2+^ binding motifs of an S100. For example, Gribenko et al. proposed that human S100P may bind Zn^2+^ in one of the Ca^2+^ binding motifs [75]. EF-hands often discriminate Ca^2+^ from Zn^2+^ and Cu^2+^, however, so this likely does not explain all of the observed transition metal binding [33,76–78].

Another feature of Zn^2+^ and Cu^2+^ binding in this family is that of variable structural responses to the same metal. Even closely related S100 proteins undergo different conformational changes when bound to a transition metal (Fig 4). This likely allows different orthologs to play different functional roles in response to transition metal binding. This can be seen for proteins that have been studied in detail. For example, human A13, which binds Cu^2+^ at a unique site, has been proposed to be involved in chaperoning Cu^2+^ as part of FGF release [29]. A9 provides another example of diverse responses to transition metals. When A9 is alone, Zn^2+^ binding is strictly necessary for one function (TLR4-activation) [79], but strongly inhibits another function (arachidnoic acid binding) [80]. This site is modified *in vivo* through the formation of a heterodimer with A8, which changes the ligating residues for one half of the site [10,81]. This creates an extremely high affinity site for Mn^2+^ and Zn^2+^ that inhibits bacterial growth by starving them of these metals [10].

Much of the transition metal binding we have observed plays no known role, but the observed binding constants (~μM) are consistent with biological concentrations of divalent transition metals. In particular, many S100 proteins are found in the extracellular space [82], where Zn^2+^ concentrations can be high enough to occupy these sites [83,84]. We expect further roles of transition metal binding to be identified in this family as it is further characterized [27], [28].

### Expansion of the family

In addition to providing insight into the evolution of transition metal binding, our phylogenetic analysis provides insight into the overall pattern of expansion of the S100 protein family. Previous phylogenies used highly incomplete taxonomic sampling and, with the exception of [36], distance-based phylogenetics [2,12,37]. We used many more sequences, from many more taxa, and applied a combined model-based/synteny analysis to better disentangle the history of the family. Our work provides support for evolutionary relationships between A13-A16, A2-A6, the calgranulins, the S100-fused proteins, and A10/A11 despite the relatively weak support for these clades taken from a purely model-based phylogenetic perspective. This also supports the previously proposed model of local gene duplication [2,12,37,48].

Our work provides evidence for earlier origins of many S100 family members than previously reported. For example, we found that the S100A2-A6 clade likely arose in ancestor of all tetrapods, and that it had the complete mammalian complement by the ancestor of amniotes. In contrast, Zimmer et al proposed this clade arose in the ancestor of mammals [2]. Some orthologs (A1, B, P, and Z) have likely been present since the last-common ancestor of vertebrates. Further, we expect that many S100 proteins actually arose even earlier than our analysis suggests. Despite having broader sampling than previous studies, our sampling of tunicates, jawless fishes, and cartilaginous fishes was still relatively sparse. Further, we relied heavily on transcriptomes, which likely underestimate the S100 complements for these organisms. As more genomic and transcriptomic datasets for these species become available, we expect to observe even earlier origins of many of the mammalian S100 orthologs.

Another difference between our tree and the published tree by Kraemer et al. [36] is that we do not see radical, parallel expansion of the S100s in bony fishes. Rather, most S100 proteins from the bony fishes are orthologous to mammalian S100s. For example, we identified 15 S100 proteins in *Takifugu rubripes* (pufferfish). All but two of them could be assigned as orthologs to human proteins (Fig 3). This said, many of these do represent lineage-specific duplications—likely via the whole genome duplications that have occurred in teleost fishes—that are co-orthologous to human proteins. The difference between our results and the previous phylogeny likely arises from our much broader sequence sampling, as the Kraemer et al. dataset was strongly biased towards sequences taken from teleosts [36].

Despite extensive taxonomic sampling, the phylogenetic tree we report is not fully resolved: the deepest branches remain obscure. This is because of the large amount of sequence divergence that has occurred between many S100 protein family members, their relatively short sequences, and the number of orthologs make full resolution of this family quite challenging. Resolution can likely be increased for individual subfamilies within the tree through even denser sampling. For example, adding further aminotes may help resolve the relationships between the amniote-specific clades identified in our analysis. We also believe increasing the sampling of amphibians would be particularly powerful, as we relied heavily on amphibian transcriptomes and likely missed S100 proteins. Better characterization of S100 proteins from amphibians may help disentangle the origins and relationships of some of the tetrapod-specific S100s (such as the calgranulins) which are, as yet, difficult to resolve. Further, signal for these relatively recent proteins could be boosted by using codon rather than amino acid substitution models.

## Conclusion

Our work reveals that transition metal binding is both ubiquitous and evolutionarily labile within the S100 protein family. Many have noted that much of the diversity of S100 function is determined by altered expression of family members [12,68,85–89]; however, our work highlights that these regulatory changes have also been accompanied by changes in sequence and biochemistry. In particular, the ease of creating and destroying transition metal binding sites has allowed rapid changes in this feature of S100 proteins. As a result, new metal binding behavior can be exploited to achieve functional diversity in the family [27,28,90], even while Ca^2+^ binding and its induced structural changes remain relatively conserved (Fig 4).

This biochemical diversification occurred rapidly during the expansion of the S100 proteins, which are a relatively young protein family. The details of how this diversification occurred are likely to encompass a rich evolutionary story. As new S100s arose via gene duplication, were they required to maintain metal binding while continuing to evolve? Or, have there been multiple cycles of loss and subsequent regain over the course of S100 evolution? What was the exact nature of metal-binding in the last common ancestor of all S100 proteins? Our observations provide groundwork to begin to ask these questions.

## Materials and Methods

### Sequence Set

We generated a database of 564 S100 protein sequences, sampled from 52 chordate species, with an emphasis on even taxonomic sampling (S1 Spreadsheet). Previous publications and preliminary database searches revealed S100 proteins were restricted to the chordates, [2,12,36] so we selected specific chordate species and characterized their S100 protein complements through extensive BLAST searches [91]. We used human proteins as seed sequences (including sentan and the S100-fused proteins, S1 Table). No published genome or transcriptome data were available for some species, so we generated de novo transcriptomes from RNAseq data in the short reads archive [92] using Trinity with default parameters [93]. The sources for our analysis are shown in S2 Table.

We removed duplicate sequences (>95% identity) from within each species using cdhit [94], and removed sequences less than 45 amino acids long. We then reverse BLAST'd all remaining sequences against the human proteome to verify they encoded S100 proteins. We aligned the sequences using msaprobs [95] followed by manual refinement in aliview [96]. Refinement was minimal and consisted of truncating variable N-terminal and C-terminal extensions, as well as several ambiguous indels. (We truncated the fused S100 protein sequences to 150 amino acids covering the S100 domain prior to alignment). The final alignment was 132 columns and had robustly aligned key columns (S1 Fig and S2 Fig, S1 Alignment).

### Phylogenetic Trees

We generated the ML tree using phyml [97] with SPR moves starting from the neighbor-joining tree. 10 random starting trees did not yield a higher likelihood tree. We found LG+Γ_8_ was the highest likelihood model [98]. We calculated aLRT-SH supports for each node [99]. In pilot analyses, the tunicate sequences were placed in random and unpredictable places on the tree (for example, coming out with mammals or in other nonsensical places on the tree). We therefore excluded them from the final ML analysis (S1 Tree).

We generated a Bayesian phylogenetic tree using Exabayes [100]. We ran two replicate MCMC runs starting from different random trees, each consisting of one main and three heated chains. We stopped the runs after 10 million generations, giving a final average split frequency of 3.97% and log likelihood ESS of 3,315. We sampled substitution models in addition to trees, giving a final 99.8% posterior probability for the JTT model [101]. We used uniform priors for all parameters. We discarded the first 15% of the trees as burn-in and generated a consensus tree by majority-rule, collapsing all nodes with posterior probabilities <50% (S2 Tree).

### Molecular cloning and Protein Expression/Purification

S100 proteins were expressed from synthesized genes in a pET28/30 vector that had an N-terminal, TEV-cleavable His tag (Millipore). Proteins were expressed in Rosetta (DE3) pLysS *E*. *coli* cells (Millipore). A saturated overnight culture was used to inoculate 1.5 L cultures at 1:150 ratio. Bacteria were grown to log-phase (OD_600_ ~ 0.8–1.0) shaking at 37°C, followed by induction of protein expression in 1 mM IPTG for ~16 hr at 16°C. Cells were harvested by centrifugation. Pellets were frozen at -20°C, where they were stored for up to 2 months. Cells were lysed by sonication in 25mM Tris, 100mM NaCl, 25mM imidazole, pH 7.4.

Primary purification was done with a 5 mL HiTrap Ni-affinity column (GE Health Science) on an Äkta PrimePlus FPLC (GE Health Science), using a 25mL gradient between 25 and 500 mM imidazole. Pooled fractions were then incubated overnight at 4°C in the presence of ~1:5 TEV protease. This cleaves the His-tag from the protein, leaving the amino acids Ser-Asn in front of the wildtype starting methionine. Proteins were further purified by hydrophobic interaction chromatography (HIC) using a 5 mL HiTrap phenyl-sepharose column (GE Health Science). This step takes advantage of the Ca^2+^-dependent exposure of a hydrophobic binding surface on the S100 proteins. Proteins were equilibrated with 2 mM CaCl_2_ and loaded onto the HIC column, followed by a 30mL gradient elution in 25mM Tris, 100mM NaCl, 5mM EDTA, pH 7.4. Proteins were then dialyzed into 4 L of 25 mM Tris, 100 mM NaCl, pH 7.4 buffer overnight at 4°C. To remove the small amount of uncleaved His-tagged protein present, proteins were then passed over another 5 mL HiTrap Ni-affinity column and the flow through collected. Finally, if any protein contaminants remained by SDS-PAGE, we performed a final anion chromatography step using a 5mL HiTrap DEAE column (GE), 25mM Tris, pH 7.0–8.5 (depending on protein) buffer with a 50mL gradient to 500 mM NaCl.

Purified proteins were dialyzed overnight against 2L of 25mM TES (or Tris), 100mM NaCl, pH 7.4, containing 2 g Chelex-100 resin (BioRad) to remove divalent metals. Purity of final protein products were >95% by SDS PAGE and MALDI-TOF mass spectrometry. Final protein products were flash frozen, dropwise, in liquid nitrogen and stored at -80°C. Typical protein yields were ~20mg/L of culture.

### Protein characterization

Prior to all biophysical measurements, we thawed and exchanged all proteins into an appropriate buffer by two serial NAP-25 desalting columns (GE Health Science). We then used A_280_ to determine protein concentration using an empirical extinction coefficient for each protein. To determine extinction coefficients, we first used ProtParam [102,103] to calculate the extinction coefficient for each protein in 6 M GdmHCl (ε_6MGdm_). We then measured the difference in A_280_ for an identical concentration of protein in native buffer versus in 6 M GdmHCl. We could then estimate a native extinction coefficient using the relationship ε_native_ = ε_6MGdm_∙A_280,native_/A_280,6MGdm_. For some proteins no correction from the predicted extinction coefficient was necessary. Extinction coefficients used for calculation of protein concentration are as follows: (hA5: 5540 M^-1^cm^-1^, hA6:5434 M^-1^cm^-1^, tunA:1490 M^-1^cm^-1^, tunB: 5699 M^-1^cm^-1^, hA2:3230 M^-1^cm^-1^, hA4:3230 M^-1^cm^-1^, hA14:7115 M^-1^cm^-1^, hA1:8480 M^-1^cm^-1^, hA11:4595 M^-1^cm^-1^, hP:2980 M^-1^cm^-1^). We also corrected for scatter in all A_280_ measurements [104].

We performed ITC experiments in 25 mM buffer, 100mM NaCl at pH 7.4 that had been chelex-treated and filtered at 0.22 μm. We selected Tris or TES as the buffering species on a case-by-case basis to ensure observable heats of binding. We equilibrated and simultaneously degassed, either by application of vacuum to the solution or by centrifugation at 18,000 x g at the experimental temperature for 60 minutes. We dissolved metals (CaCl_2_, ZnCl_2_, or CuCl_2_) directly into the experimental buffer immediately prior to each experiment. We performed all experiments at 25°C using a MicroCal ITC-200 or a MicroCal VP-ITC (GE Health Sciences). Data were collected using low gain or no gain, with 750 rpm syringe stir speed. Shot spacing ranged from 120s-2400s depending on gain settings and relaxation time of the binding process. These setting were optimized on a per protein basis. Data were fit to one or two site models using the Origin 7 software. For binding curves with obvious 1:1 stoichiometry the one-site model in Origin was used. For data with apparent 2:1 stoichiometry, evident from location of inflection points in the data, a fit of the included two-site model was attempted. If the two-site model could not be fit, we then used a single-site binding model with a floating stoichiometry to extract an apparent binding constant across sites.

We collected far-UV circular dichroism data between 200–250 nm using a J-815 CD spectrometer (Jasco) with a 1 mm quartz spectrophotometer cell (Starna Cells, Inc. Catalog No. 1-Q-1). We prepared 20–50 μM samples in a TES buffer identical to that used for ITC. We centrifuged at 18,000 x g in a temperature-controlled centrifuge at the experimental temperature prior to experiments. We collected 5 scans for each condition, and then averaged the spectra and subtracted a blank buffer spectrum using the Jasco spectra analysis software suite. We converted raw ellipticity into mean molar ellipticity using the concentration and number of residues in each protein. We collected intrinsic tyrosine and/or tryptophan fluorescence using a J-815 CD spectrometer (Jasco) with an attached model FDT-455 fluorescence detector (Jasco) using a 1 cm quartz cuvette (Starna Cells, Inc.). We prepared 5–20 μM samples exactly as we did for our CD experiments. We collected 3–5 replicate scans for each condition, and then averaged the spectra and subtracted a blank buffer spectrum (averaged from 10–15 buffer blank spectra) using the Jasco spectra analysis software suite. For all spectroscopic measurements, we verified the reversibility of metal-induced changes to the spectra by measuring the apo spectrum, adding the appropriate metal and re-measuring the spectrum, and then adding excess EDTA and re-measuring the spectrum.

### Native electrospray ionization time-of-flight mass spectrometry (nano ESI-MS)

To prepare samples for mass spectrometry experiments small (~200uL) samples of the proteins used in MS experiments were dialyzed for at least 24 hr against 2–4 L of either 10 or 100mM ammonium acetate, pH 7.4 to remove salt and exchange into a more optimal buffer for MS. Samples were then diluted to ~10uM in the dialysis buffer prior to experiment. All mass spectra were acquired using a Waters Synapt G2-Si ion-mobility mass spectrometer equipped with a nanoelectrospray (nanoESI) source and operated in “Sensitivity” mode. NanoESI emitters were pulled from borosilicate capillaries (ID 0.78 mm) to a tip ID of approximately 1 μm using a Sutter Flaming-Brown P-97 micropipette puller. 3–5 μL of sample were loaded into an emitter, a platinum wire was placed in electrical contact with the solution, and a potential of +0.8–1.2 kV was applied to the wire to initiate electrospray. The source temperature was equilibrated to ambient temperature, trap and transfer collision voltages were set to 25 V and 5 V, respectively, and the trap gas used was argon at a flow rate of 5 mL/min. Reported spectra are the sum of ~3 minutes of continuously-collected data. Mass calibration was achieved using the series of Cs(CsI)_n_^1+^ peaks produced from nanoESI of 0.1 M aqueous cesium iodide (Aldrich).

We carefully controlled for spurious dimers in our nanoESI-MS experiments. Non-specific dimers (and high-order oligomers) can arise if, by chance, more than one monomer ends up in an electrospray drop. These non-specific aggregates are expected to follow a roughly Poisson distribution of oligomeric states, governed by the bulk concentration of monomers in solution. These non-specific species can be distinguished from specific oligomeric species by measuring the mass spectrum over a wide range of protein concentrations. Dimers observed at 10 μM could be the result of non-specific interactions; dimers observed at 10 nM are almost certainly not. This can be seen by considering the distribution of non-specific species across drops. Under our instrumental conditions, electrospray creates drops ~100–200 nm in diameter, meaning that 10 nM protein solution will yield drops that contain, on average, ~0.003–0.025 protein molecules. Taking the upper limit of 0.025 protein molecules per drop, one would expect only 0.2% of drops to have non-specific dimers. Increasing to 100 nM protein takes this to 2.4% of drops. If one goes to 1 μM, non-specific dimers become quite significant (25.6%), but this is accompanied by a large number of non-specific trimers (21.4%). Although many factors, including relative ionization efficiency and instrumental conditions, can affect the observed abundances of ions formed from electrospray, these effects should be largely independent of initial solution concentration under the instrumental conditions used here.

We interpreted the mass spectra shown in Fig 5D and S14 using this logic. Mass spectra of proteins at low concentrations (10–100 nM) exhibit unexpectedly abundant monomers and dimers, consistent with a specific dimer. Mass spectra at high concentrations (1–10 μM) exhibit dimers but not trimers, again consistent with a specific dimer rather than non-specific, Poisson-governed aggregation in drops. The small population of tetramer for tunB at 10 μM could either reflect a true tetramer or a random partitioning of two dimers into an electrospray drop at this high concentration.

### Sedimentation velocity analytical ultracentrifugation

Samples were concentrated to ~50uM and then dialyzed against 2L of 25 mM TES, 100mM NaCl, 1mM TCEP, pH 7.4) overnight at 4°C using 6000–80000 MWCO dialysis tubing. Prior to sedimentation velocity experiments proteins were then centrifuged at >18000 x g for 30 min. in a temperature-controlled centrifuge. AUC experiments were performed at 50k x g in sector-shaped cells with sapphire windows (Beckman) on a Beckman ProteomeLab XL-1 analytical ultracentrifuge. Due to the low extinction coefficients of the proteins, sedimentation was monitored using interference mode rather than absorbance at 280nm. Sedimentation velocity data was fit numerically to the Lamm equation and the c(s) distribution determined using SedFit [105,106]. Estimated sedimentation coefficients and molecular masses of species present in solution were calculated from the fits.

### Homology model

The homology model of tunB was constructed using Modeller 9.17 [107] using 46 Ca^2+^ bound crystal structures (without bound peptide targets) as combined templates (PDB:1e8a,1gqm,1j55,1k96,1k9k,1mho,1mr8,1odb,1qlk,1xk4,1xyd,1yut,1yuu,1zfs,2egd,2h2k,2h61,2k7o,2kay,2l51,2psr,2q91,2wnd,2wor,2wos,2y5i,3c1v,3cga,3cr2,3cr4,3cr5,3czt,3d0y,3d10,3gk1,3gk2,3gk4,3hcm,3icb,3iqo,3lk0,3lk1,3lle,3m0w,3psr,3rlz, and 4duq). Alignment was generated using the PAIRWISE alignment method with default parameters. Model was generated as a dimer, with the single tunicate sequence mapped to both the A and B chains. Automodel was used to generate models, using default parameters. 20 models were generated and the best selected by DOPE score. The final model had an RMSD of 0.65 Å^2^ relative to the crystal structure of S100B bound to Ca^2+^ and Zn^2+^ (PDB: 3czt).

## Supporting Information

S1 Alignment Alignment used for Bayesian and ML tree construction. Alignment is in fasta format, using names described in S1 Spreadsheet for each sequence.
(TXT)

S1 Fig Sequence logo of alignment of S100 proteins. Sequence logo indicates relative frequency of amino acids at each position in the alignment. Taller letters indicate higher frequency at that position. Arrows indicate 13 key residues we used to verify/anchor the alignment.
(PDF)

S2 Fig Graphical representation of sequence alignment of S100 proteins used in this analysis. Colored characters indicate characters that match the consensus for the column. Arrows indicate the key residues highlighted in S1 Fig. Sequence names are as in S1 Spreadsheet.
(PDF)

S3 Fig Bayesian consensus tree of 564 S100 proteins drawn from 52 Olfactores species. Tree is a majority rule consensus tree, with all nodes with posterior probabilities <50% collapsed into polytomies. Wedges are collapsed clades of shared orthologs, with wedge height denoting number of included taxa and wedge length denoting longest branch length with the clade. Support values are posterior probabilities. Rooting is arbitrary given the poor resolution at the base of the taxonomic tree. Icons indicate taxonomic classes represented within each clade: tunicates (black sea squirt), jawless fishes (pink lamprey), cartilaginous fishes (purple ray), ray-finned fishes (light blue fish), lobe-finned fishes (blue coelacanth), amphibans (green frog), birds/reptiles (yellow lizard), and mammals (red mouse). Inset shows estimated divergence times for each taxonomic class in millions of years before present.
(PDF)

S4 Fig Example ITC traces for various S100 proteins. Each panel is a single human paralog, indicated by the name on the graph. Color of fit indicates metal used as titrant: Zn^2+^(gray) or Cu^2+^ (copper). Top sub-panel for each panel is a raw power vs. time curve. Bottom sub-panel for each panel is integrated heat versus molar ratio. The model fit is denoted by the heavy line through the fit points.
(PDF)

S5 Fig Human S100 paralogs exhibit different structural changes in response to Zn^2+^ and Ca^2+^. Curves are far-UV CD spectra (mean molar ellipticity vs. wavelength). Colors represent metal: apo (black), Zn^2+^ (gray), and Ca^2+^ (blue). Paralog is indicated to the right of each spectrum.
(PDF)

S6 Fig Biophysical characterization of tunA. A) ITC trace for binding of Ca^2+^. B) ITC trace for binding of Zn^2+^. C) Far-UV CD spectra for tunA in apo form (black), presence of Ca^2+^ (blue) and presence of Zn2+ (gray). D) Intrinsic fluorescence spectra for tunA with conditions as in panel C. E-H) ESI-MS spectra for tunA, titrating from 10 μM to 0.01 μM protein. Icons indicate species (monomer or dimer). Numbers indicate charge state. Dimer is lost preferentially during dilution, suggesting it is an artifact of electrospray process.
(PDF)

S7 Fig tunB dilution by ESI-MS. tunB mass spectra at concentrations of a) 10 μM, b) 1 μM, c) 0.1 μM, and d) 0.01 μM demonstrate that tunB homodimers are robust to dilution, indicating that this is a specific interaction. Homotetramer is observed only in the most concentrated sample, thus homotetramer signal likely arises from non-specific interactions during the electrospray process.
(PDF)

S8 Fig Tunciate S100s form homodimers in sedimentation velocity experiments. Graph shows the distribution of sedimentation coefficient determined for tunA (black) and tunB (blue). The apparent mass of the homodimer peaks are indicated above each peak, with the mass expected from the amino acid sequence of the protein in parentheses.
(PDF)

S1 Spreadsheet Database of sequences used for phylogenetic analysis. Columns are: *ortholog*: orthology call, relative to human proteins; *common name*: common name of organism; *name in alignment/tree*: unique name assigned to sequence in the alignment and tree files included in the supplemental materials; *scientific name*: scientific name of organism; *database*: database from which sequence was extracted; *sequence id*: accession number or internal, unique identifier of each sequence; *protein sequence*: sequence of the S100 protein used.
(XLS)

S1 Table Human S100 sequences used for BLAST. (PDF)

S2 Table Sources and accession numbers for all sequences used in phylogenetics analysis. Contains the database used for each species, as well as relevant citations to primary literature.
(PDF)

S3 Table Thermodynamic parameters for metal binding to S100 proteins, determined by ITC. (PDF)

S1 Tree Maximum-likelihood tree for S100 protein family. Tree is in newick format. Sequence names are as in S1 Spreadsheet. Support values are aLRT/SH supports.
(TXT)

S2 Tree Bayesian consensus tree for S100 protein family. Tree is in newick format. Sequence names are as in S1 Spreadsheet. Consensus was created by majority-rule, collapsing all nodes with posterior probabilities <50%. Support values are posterior probabilities for last 85% trees in MCMC runs.
(TXT)
