# Supplementary material for: Multiple Evolutionary Origins of Ubiquitous Cu2+ and Zn2+ Binding in the S100 Protein Family
Source: PLoS One. 2016 Oct 20;11(10):e0164740. doi: 10.1371/journal.pone.0164740 (PMC5072561; doi:10.1371/journal.pone.0164740)
Supplement: S1 Fig — Sequence logo indicates relative frequency of amino acids at each position in the alignment. Taller letters indicate higher frequency at that position. Arrows indicate 13 key residues we used to verify/anchor the alignment. (PDF) [file pone.0164740.s002.pdf]

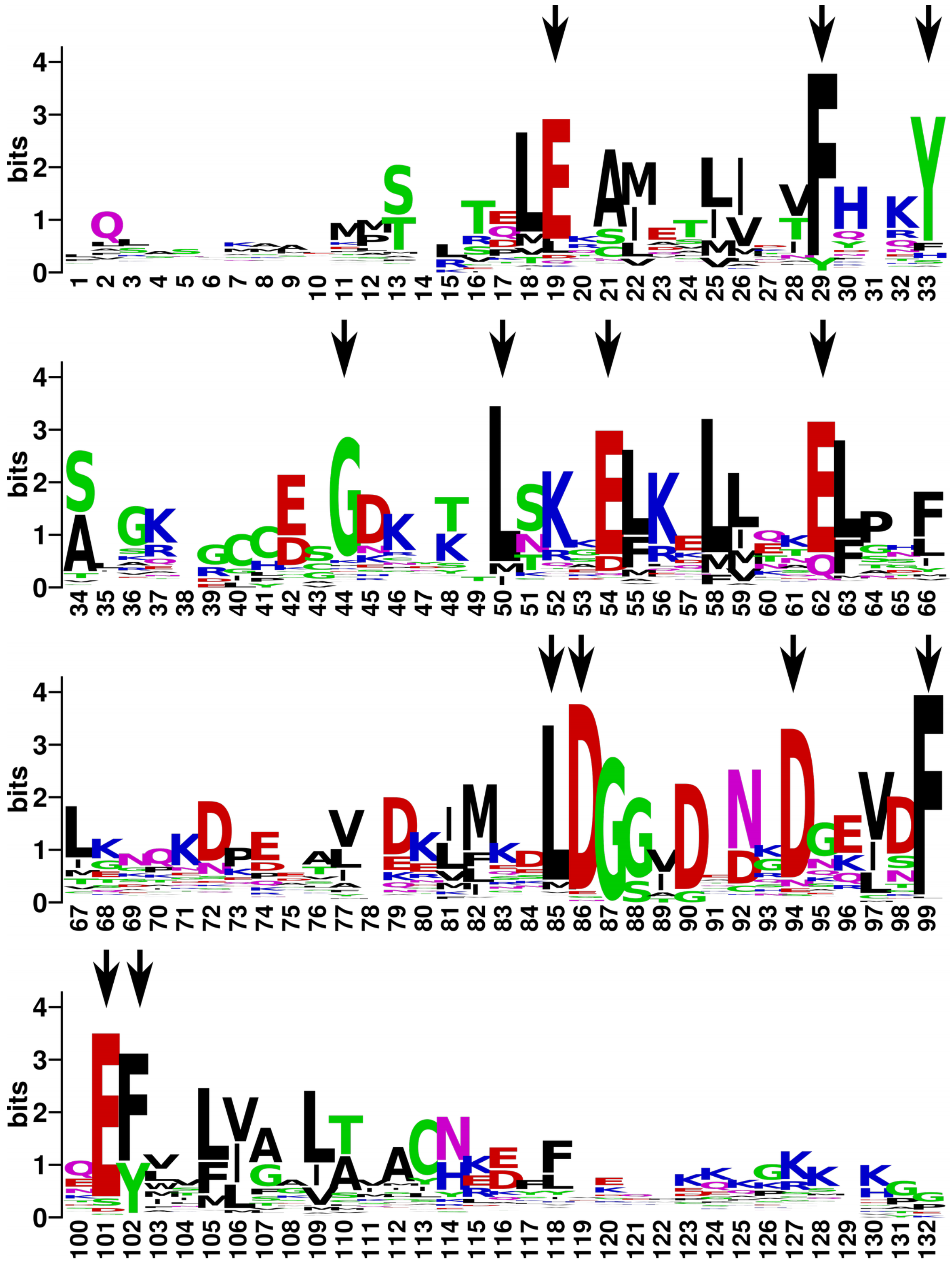

S1 Fig: Sequence logo of alignment of S100 proteins. Sequence logo indicates relative occurrence of amino acids at each position in the alignment. Taller letters indicate higher frequency at that position. Arrows indicate 13 key residues we used to verify/anchor the alignment.
