# Supplementary material for: Multiple Evolutionary Origins of Ubiquitous Cu2+ and Zn2+ Binding in the S100 Protein Family
Source: PLoS One. 2016 Oct 20;11(10):e0164740. doi: 10.1371/journal.pone.0164740 (PMC5072561; doi:10.1371/journal.pone.0164740)
Supplement: S3 Fig — Tree is a majority rule consensus tree, with all nodes with posterior probabilities <50% collapsed into polytomies. Wedges are collapsed clades of shared orthologs, with wedge height denoting number of included taxa and wedge length denoting longest branch length with the clade. Support values are posterior probabilities. Rooting is arbitrary given the poor resolution at the base of the taxonomic tree. Icons indicate taxonomic classes represented within each clade: tunicates (black sea squirt), jawless fishes (pink lamprey), cartilaginous fishes (purple ray), ray-finned fishes (light blue fish), lobe-finned fishes (blue coelacanth), amphibans (green frog), birds/reptiles (yellow lizard), and mammals (red mouse). Inset shows estimated divergence times for each taxonomic class in millions of years before present. (PDF) [file pone.0164740.s004.pdf]

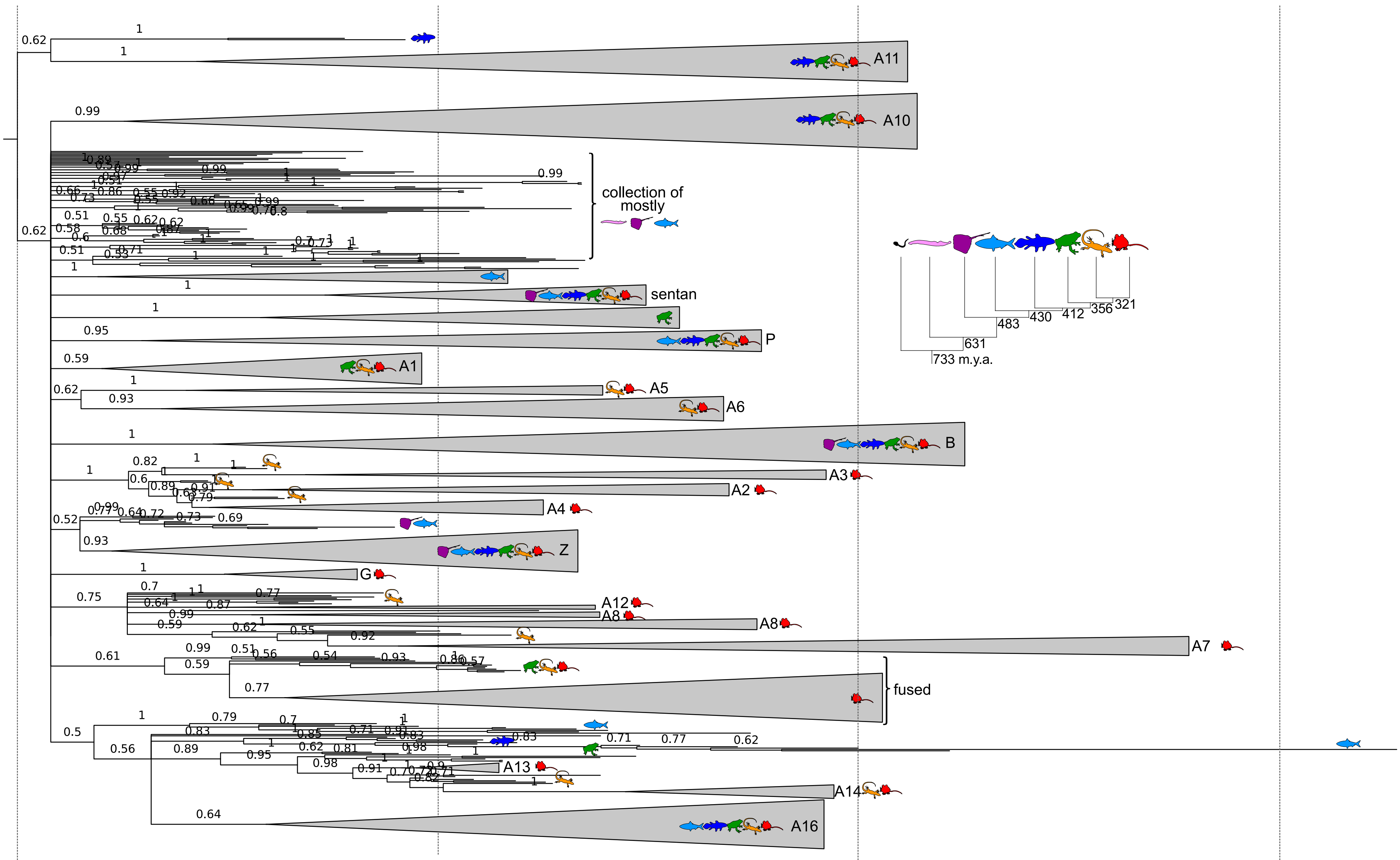

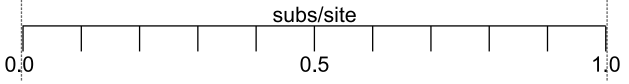

Fig S3: Bayesian consensus tree of 564 S100 proteins drawn from 52 Olfactores species. Tree is a majority rule consensus tree, with all nodes with posterior probabilities <50% collapsed into polytomies. Wedges are collapsed clades of shared orthologs, with wedge height denoting number of included taxa and wedge length denoting longest branch length with the clade. Support values are posterior probabilities. Rooting is arbitrary given the poor resolution at the base of the taxonomic tree. Icons indicate taxonomic classes represented within each clade: tunicates (black sea squirt), jawless fishes (pink lamprey), cartilaginous fishes (purple ray), ray-finned fishes (light blue fish), lobe-finned fishes (blue coelacanth), amphibans (green frog), birds/reptiles (yellow lizard), and mammals (red mouse). Inset shows estimated divergence times for each taxonomic class in millions of years before present.
