# Supplementary material for: Multiple Evolutionary Origins of Ubiquitous Cu2+ and Zn2+ Binding in the S100 Protein Family
Source: PLoS One. 2016 Oct 20;11(10):e0164740. doi: 10.1371/journal.pone.0164740 (PMC5072561; doi:10.1371/journal.pone.0164740)
Supplement: S4 Fig — Each panel is a single human paralog, indicated by the name on the graph. Color of fit indicates metal used as titrant: Zn2+(gray) or Cu2+ (copper). Top sub-panel for each panel is a raw power vs. time curve. Bottom sub-panel for each panel is integrated heat versus molar ratio. The model fit is denoted by the heavy line through the fit points. (PDF) [file pone.0164740.s005.pdf]

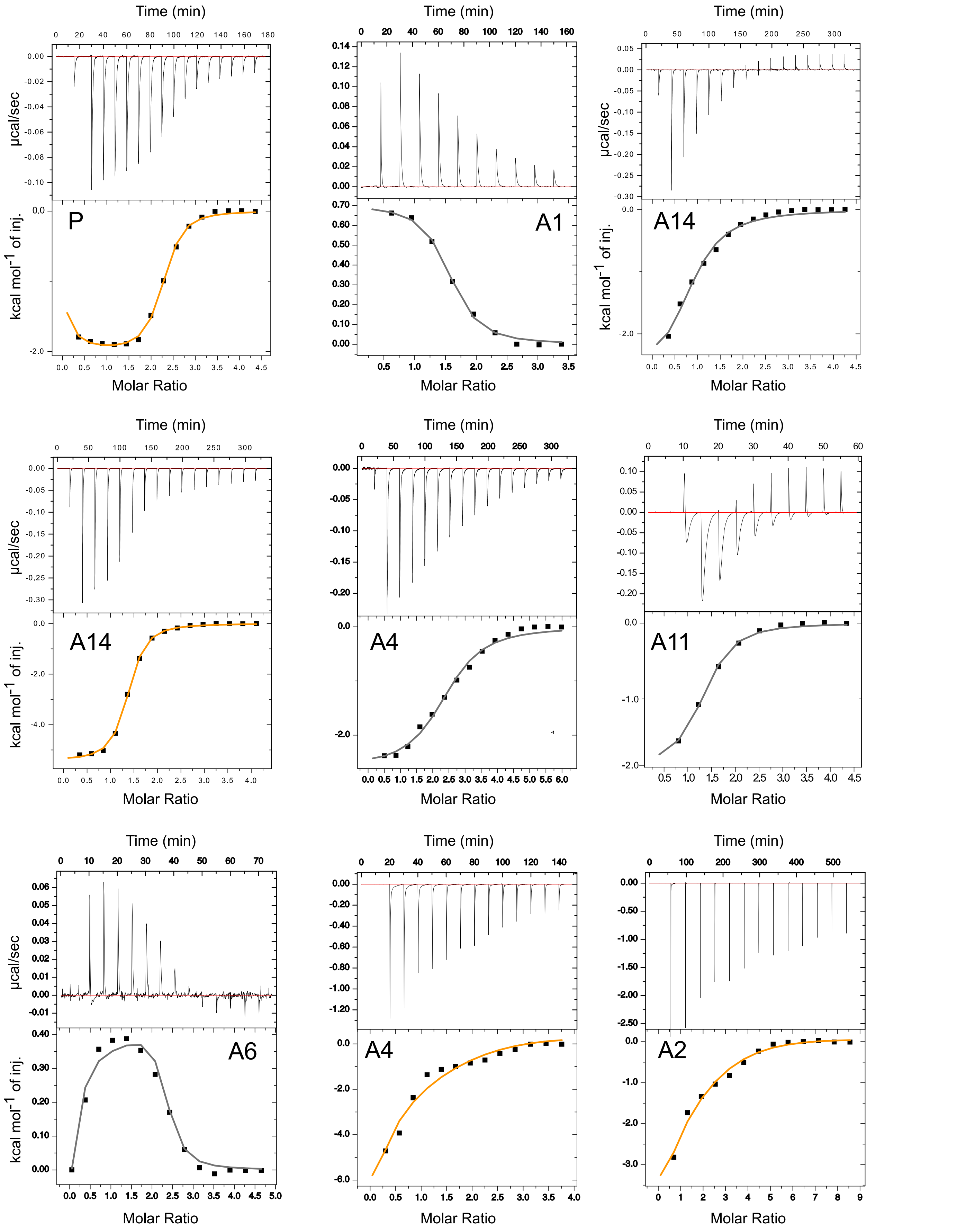

S4 Fig: Example ITC traces for various S100 proteins. Each panel is a single human ortholog, indicated by the name on the graph. Color of fit indicates metal used as titrant: Zn^{2+} (gray) or Cu^{2+} (copper). Top sub-panel for each panel is a raw power vs. time curve. Bottom sub-panel for each panel is integrated heat versus molar ratio. The model fit is denoted by the heavy line through the fit points.
