# Supplementary material for: Multiple Evolutionary Origins of Ubiquitous Cu2+ and Zn2+ Binding in the S100 Protein Family
Source: PLoS One. 2016 Oct 20;11(10):e0164740. doi: 10.1371/journal.pone.0164740 (PMC5072561; doi:10.1371/journal.pone.0164740)
Supplement: S5 Fig — Curves are far-UV CD spectra (mean molar ellipticity vs. wavelength). Colors represent metal: apo (black), Zn2+ (gray), and Ca2+ (blue). Paralog is indicated to the right of each spectrum. (PDF) [file pone.0164740.s006.pdf]

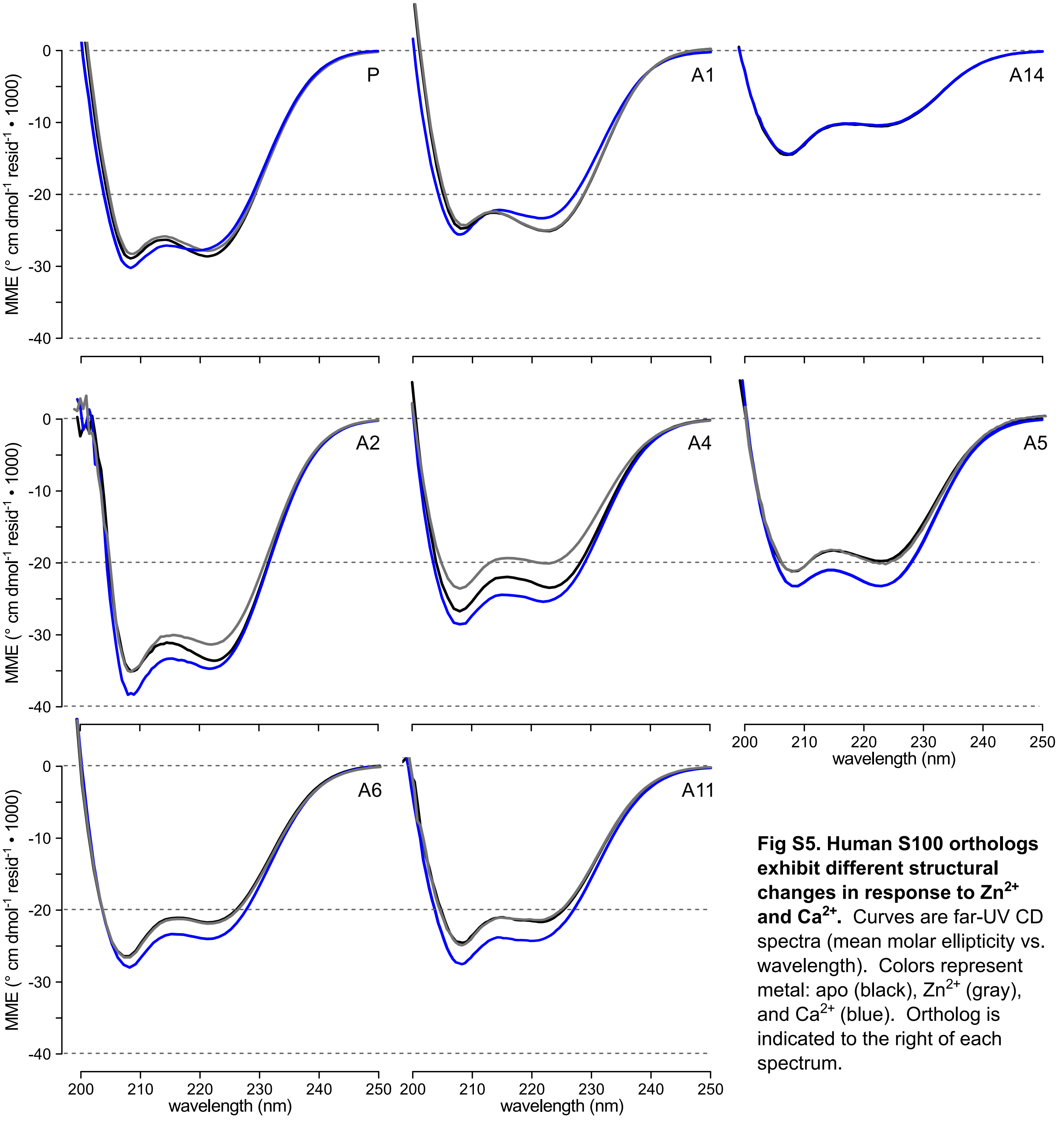

Fig S5. Human S100 orthologs exhibit different structural changes in response to Zn^{2+} and Ca^{2+}. Curves are far-UV CD spectra (mean molar ellipticity vs. wavelength). Colors represent metal: apo (black), Zn^{2+} (gray), and Ca^{2+} (blue). Ortholog is indicated to the right of each spectrum.
