# Supplementary figures and images for: Multiple Evolutionary Origins of Ubiquitous Cu2+ and Zn2+ Binding in the S100 Protein Family
Source: PLoS One. 2016 Oct 20;11(10):e0164740. doi: 10.1371/journal.pone.0164740 (PMC5072561; doi:10.1371/journal.pone.0164740)

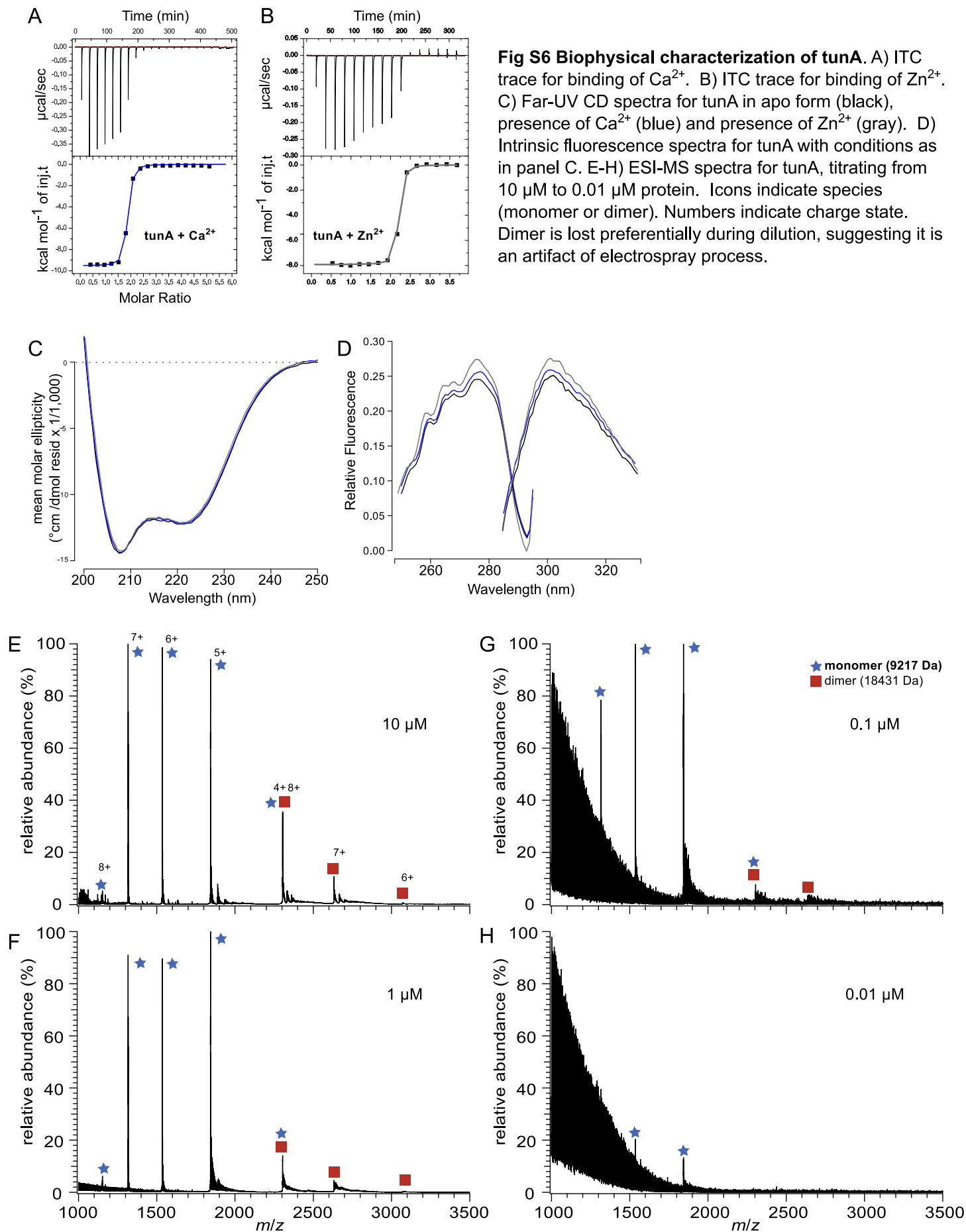

Supplement: S6 Fig — A) ITC trace for binding of Ca2+. B) ITC trace for binding of Zn2+. C) Far-UV CD spectra for tunA in apo form (black), presence of Ca2+ (blue) and presence of Zn2+ (gray). D) Intrinsic fluorescence spectra for tunA with conditions as in panel C. E-H) ESI-MS spectra for tunA, titrating from 10 μM to 0.01 μM protein. Icons indicate species (monomer or dimer). Numbers indicate charge state. Dimer is lost preferentially during dilution, suggesting it is an artifact of electrospray process. (PDF) [file pone.0164740.s007.pdf]
