# Supplementary material for: Multiple Evolutionary Origins of Ubiquitous Cu2+ and Zn2+ Binding in the S100 Protein Family
Source: PLoS One. 2016 Oct 20;11(10):e0164740. doi: 10.1371/journal.pone.0164740 (PMC5072561; doi:10.1371/journal.pone.0164740)
Supplement: S7 Fig — tunB mass spectra at concentrations of a) 10 μM, b) 1 μM, c) 0.1 μM, and d) 0.01 μM demonstrate that tunB homodimers are robust to dilution, indicating that this is a specific interaction. Homotetramer is observed only in the most concentrated sample, thus homotetramer signal likely arises from non-specific interactions during the electrospray process. (PDF) [file pone.0164740.s008.pdf]

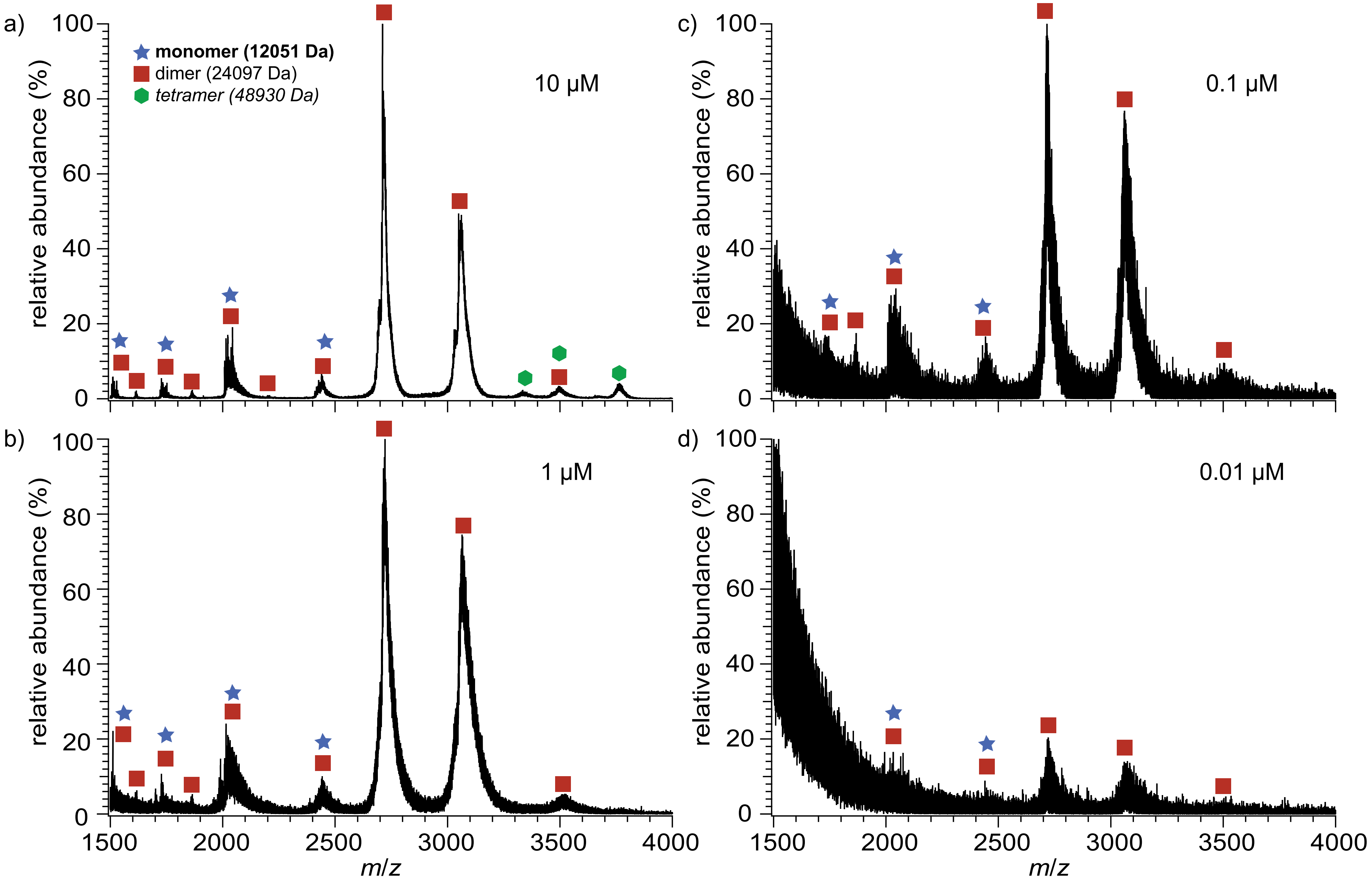

S7 Fig tunB dilution by ESI-MS. tunB mass spectra at concentrations of a) 10 µM, b) 1 µM, c) 0.1 µM, and d) 0.01 µM demonstrate that tunB homodimers are robust to dilution, indicating that this is a specific interaction. Homotetramer is observed only in the most concentrated sample, thus homotetramer signal likely arises from non-specific interactions during the electrospray process.
