# Supplementary material for: Multiple Evolutionary Origins of Ubiquitous Cu2+ and Zn2+ Binding in the S100 Protein Family
Source: PLoS One. 2016 Oct 20;11(10):e0164740. doi: 10.1371/journal.pone.0164740 (PMC5072561; doi:10.1371/journal.pone.0164740)
Supplement: S8 Fig — Graph shows the distribution of sedimentation coefficient determined for tunA (black) and tunB (blue). The apparent mass of the homodimer peaks are indicated above each peak, with the mass expected from the amino acid sequence of the protein in parentheses. (PDF) [file pone.0164740.s009.pdf]

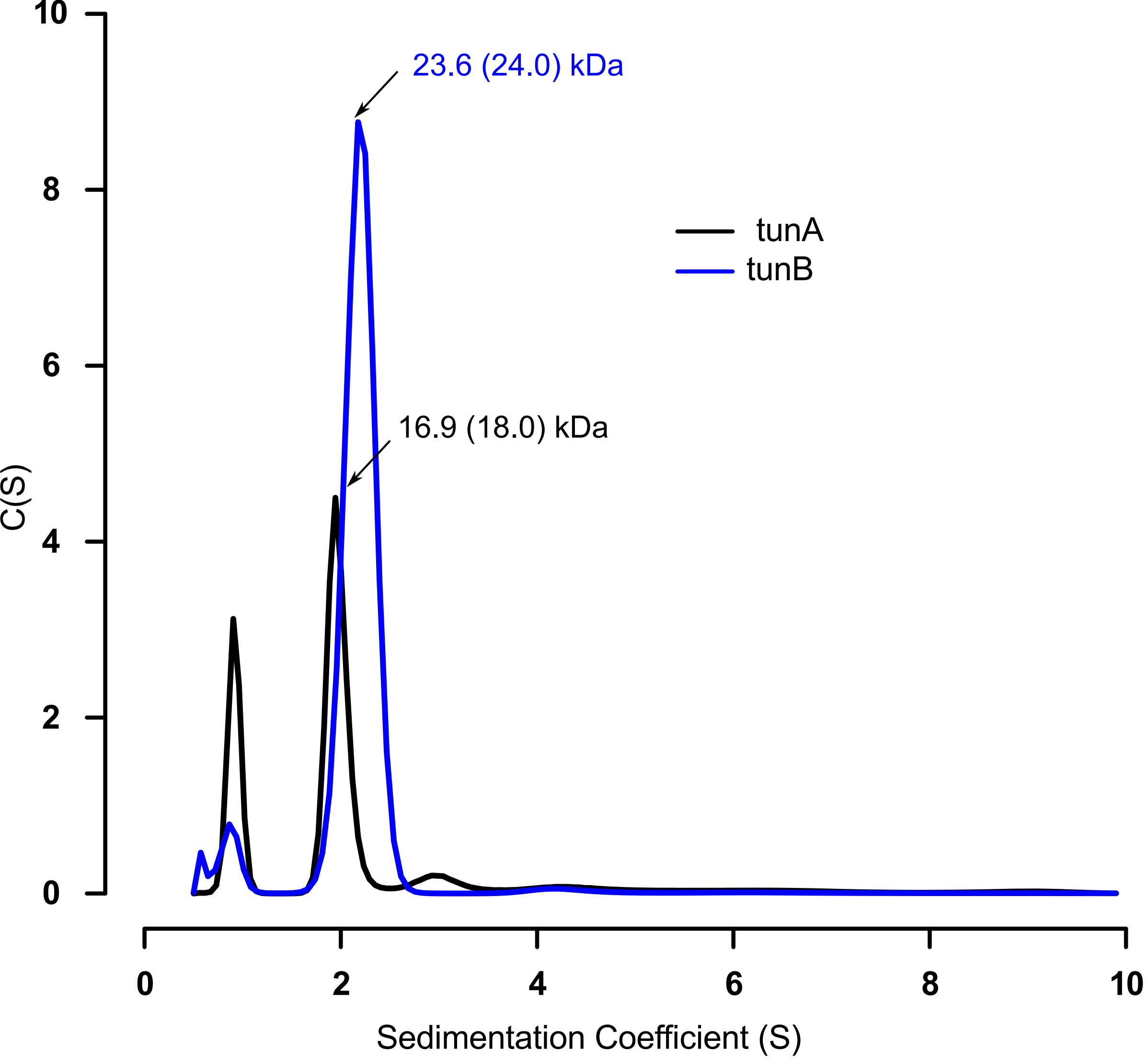

S8 Fig: Tunciate S100s form homodimers in sedimentation velocity experiments. Graph shows the distribution of sedimentation coefficient determined for tunA (black) and tunB (blue). The apparent mass of the homodimer peaks are indicated above each peak, with the mass expected from the amino acid sequence of the protein in parentheses.
