# Supplementary material for: Multiple Evolutionary Origins of Ubiquitous Cu2+ and Zn2+ Binding in the S100 Protein Family
Source: PLoS One. 2016 Oct 20;11(10):e0164740. doi: 10.1371/journal.pone.0164740 (PMC5072561; doi:10.1371/journal.pone.0164740)
Supplement: S1 Table — (PDF) [file pone.0164740.s011.pdf]

S1 Table: Human S100 sequences used for BLAST

uniprot	protein	protein length	class	chromosome
P23297	S100A1	94	S100	1
P29034	S100A2	98	S100	1
P33764	S100A3	101	S100	1
P26447	S100A4	101	S100	1
P33763	S100A5	92	S100	1
P06703	S100A6	90	S100	1
P31151	S100A7	101	S100	1
Q86SG5	S100A7A	101	S100	1
P05109	S100A8	93	S100	1
P06702	S100A9	114	S100	1
P60903	S100A10	97	S100	1
P31949	S100A11	105	S100	1
P80511	S100A12	92	S100	1
Q99584	S100A13	98	S100	1
Q9HCY8	S100A14	104	S100	1
Q96FQ6	S100A16	103	S100	1
P04271	S100B	92	S100	21
P29377	S100G	79	S100	X
P25815	S100P	95	S100	1
Q8WXG8	S100Z	99	S100	5
Q9UBG3	Cornulin	495	fused S100	1
P20930	Filaggrin	4061	fused S100	1
Q5D862	Filaggrin-2	2391	fused S100	1
Q86YZ3	Hornerin	2850	fused S100	1
Q6XPR3	Repetin	784	fused S100	1
A6NMZ2	Sentan	147	S100	3
Q07283	Trichohyalin	1943	fused S100	1
