# Supplementary material for: Multiple Evolutionary Origins of Ubiquitous Cu2+ and Zn2+ Binding in the S100 Protein Family
Source: PLoS One. 2016 Oct 20;11(10):e0164740. doi: 10.1371/journal.pone.0164740 (PMC5072561; doi:10.1371/journal.pone.0164740)

Fig S2: Sequence alignment for S100 proteins used in this analysis. Colored characters indicate characters that match the consensus for the column. Arrows indicate the key residues highlighted in Fig S1.

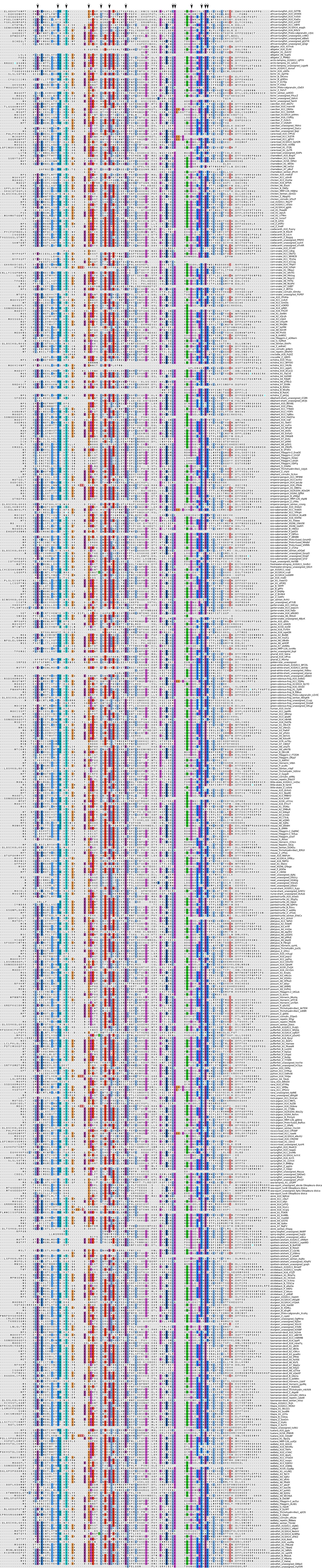

Supplement: S2 Fig — Colored characters indicate characters that match the consensus for the column. Arrows indicate the key residues highlighted in S1 Fig. Sequence names are as in S1 Spreadsheet. (PDF) [file pone.0164740.s003.pdf]
