# Supplementary material for: Multiple Evolutionary Origins of Ubiquitous Cu2+ and Zn2+ Binding in the S100 Protein Family
Source: PLoS One. 2016 Oct 20;11(10):e0164740. doi: 10.1371/journal.pone.0164740 (PMC5072561; doi:10.1371/journal.pone.0164740)
Supplement: S3 Table — (PDF) [file pone.0164740.s013.pdf]

	homolog	$K_{D,1}$ (μM)	ΔH_1 (kcal•mol^{-1})	n (#)	$K_{D,2}$ (μM)	ΔH_2 (kcal•mol^{-1})	fit model	buffer	# reps
Zn^{2+}	P	2.7 ± n/a	-0.4 ± n/a	0.7 ± n/a	---	---	equivalent sites	Tris	1
	A1	3.5 ± 0.7	0.8 ± 0.1	1.3 ± 0.2	---	---	equivalent sites	TES	2
	A14	27.9 ± 12.6	-2.3 ± 0.5	1.0 ± 0.2	---	---	equivalent sites	Tris	2
	A2	33.2 ± 2.3	6.1 ± 1.4	2 ± n/a	83.4 ± 2.1	-9.1 ± 1.1	independent sites	Tris	2
	A4	12.5 ± 1.3	-4.9 ± 2.3	2.2 ± 0.1	**	**	equivalent sites	Tris	2
	A5	1.3 ± 0.3	-1.4 ± 0.4	0.9 ± 0.1	---	---	equivalent sites	Tris	2
	$A5_{H17A}$	3.0 ± 0.1	-2.5 ± 0.2	0.8 ± 0.1	---	---	equivalent sites	Tris	2
	$A5_{C79S}$	---	---	---	---	---	equivalent sites	Tris	2
	$A5_{C43S/C79S}$	---	---	---	---	---	equivalent sites	Tris	2
	A6	**	**	**	**	**	**	Tris	2
	A11	10.9 ± 0.3	-2.0 ± 0.1	1.2 ± 0.1	---	---	equivalent sites	Tris	2
	tunA	0.1 ± 0.1	-7.4 ± 0.5	2.0 ± 0.1	**	**	equivalent sites	TES	2
	tunB	2.6 ± n/a	8.2 ± n/a	1.1 ± n/a	---	---	equivalent sites	TES	1
Cu^{2+}	P	1.7 ± 0.8	-2.4 ± 0.3	2.1 ± 0.1	**	**	equivalent sites	TES	2
	A1	---	---	---	---	---	---	TES	2
	A14	1.1 ± 0.3	-5.2 ± 0.2	1.5 ± 0.2	---	---	equivalent sites	TES	2
	A2	**	**	2 ± n/a	**	**	**	TES	2
	A4	**	**	2 ± n/a	**	**	**	TES	2
	A5	1.2 ± 0.1	-3.6 ± 0.2	1.2 ± 0.1	---	---	equivalent sites	TES	2
	$A5_{H17A}$	2.9 ± 1.2	-4.3 ± 0.6	1.1 ± 1.4	---	---	equivalent sites	TES	2
	$A5_{C79S}$	6.4 ± 0.2	-3.1 ± 0.1	0.9 ± 0.1	---	---	equivalent sites	TES	2
	$A5_{C43S/C79S}$	2.4 ± 0.6	-2.6 ± 0.2	1.2 ± 0.1	---	---	equivalent sites	TES	2
	A6	1.9 ± 0.5	-3.6 ± 0.3	0.8 ± 0.1	---	---	equivalent sites	TES	2
	A11	2.1 ± 0.1	-5.4 ± 0.2	2.4 ± 0.1	---	---	equivalent sites	TES	2
Ca^{2+}	tunA	0.1 ± 0.1	-9.5 ± 1.6	2.1 ± 0.2	**	**	equivalent sites	TES	2
	tunB	1.6 ± 0.3	246 ± 86	1.8 ± 0.2	1.6 ± 0.3	-133 ± 61	equivalent sites	TES	2
