# Supplementary material for: Multiple Evolutionary Origins of Ubiquitous Cu2+ and Zn2+ Binding in the S100 Protein Family
Source: PLoS One. 2016 Oct 20;11(10):e0164740. doi: 10.1371/journal.pone.0164740 (PMC5072561; doi:10.1371/journal.pone.0164740)
Supplement: S2 Table — Contains the database used for each species, as well as relevant citations to primary literature. (PDF) [file pone.0164740.s012.pdf]

S2 Table: Sequence sources

source	citation	species
Uniprot<br><br>Genome	(Consortium 2015)	*Bos taurus*<br>*Gallus gallus*<br>*Homo sapiens*<br>*Loxodonta africana*<br>*Monodelphis domestica*<br>*Mus musculus*<br>*Ornithorhynchus anatinus*<br>*Sarcophilus harrisii*
Ensembl v.84 predicted peptides<br><br>Genome	(Kersey et al. 2016)	*Danio rerio*<br>*Gadus morhua*<br>*Gasterosteus aculeatus*<br>*Latimeria chalumnae*<br>*Lepisosteus oculatus*<br>*Macropus eugenii*<br>*Oreochromis niloticus*<br>*Takifugu rubripes*
reptilian-transcriptomes.org<br><br>Transcriptome	(Tzika et al. 2015)	*Alligator mississippiensis*<br>*Chalcides ocellatus*<br>*Chamaeleo chamaeleon*<br>*Chrysemys picta*<br>*Crocodylus porosus*<br>*Eublepharis macularius*<br>*Gavialis gangeticus*<br>*Pantherophis guttatus*<br>*Python molurus bivittatus*<br>*Sphenodon punctatus*<br>*Thamnophis elegans*
Avianbase<br>Genome	(Eöry et al. 2015)	*Aptenodytes forsteri*<br>*Columba livia*
Skatebase<br>Genome	(Wang et al. 2012)	*Callorhinchus milii*<br>*Leucoraja erinacea*<br>*Scyliorhinus canicula*
SRA transcriptomes<br>(Leinonen et al. 2011)	SRR1693777<br>SRR1604859<br>SRR652971<br>SRR1693776<br>SRR1693787<br>SRR1695991<br>SRR388685	*Acipenser transmontanus*<br>*Botryllus schlosseri*<br>*Ginglymostoma cirratum*<br>*Ichthyophis bannanicus*<br>*Lepidosiren paradoxa*<br>*Lethenteron camtschaticum*<br>*Petromyzon marinus*

	SRR1612395	*Polypterus senegalus*
	SRR2039259	*Potamotrygon amandae*
	SRR2028021	*Protopterus annectens*
	SRR1514129	*Scyliorhinus canicula*
	SRR931704	*Tachyglossus aculeatus*
Published transcriptomes	(Richards et al. 2013)	*Carcharodon carcharias*
	(Matsunami et al. 2015b, 2015a)	*Hynobius retardatus*
	(Robertson and Cornman 2014, 2013)	*Lithobates clamitans*
	(Qiao et al. 2013)	*Odorrana margaretae*
	(Wang et al. 2015)	*Oikopleura dioica*
	(Genomic Resources Development Consortium et al. 2015; Nourisson et al. 2014)	*Rhinella marina*
	(Genomic Resources Development Consortium et al. 2015; Nourisson et al. 2014)	*Rhinella schneideri*
	(Deck et al. 2013)	*Squalus acanthias*
	(B. Wielstra et al. 2014; Ben Wielstra et al. 2014)	*Triturus cristatus*

Works Cited

Consortium, T.U. 2015. UniProt: a hub for protein information. *Nucleic Acids Res.* **43**: D204–D212.

Deck, C.A., McKay, S.J., Fiedler, T.J., LeMoine, C.M.R., Kajimura, M., Nawata, C.M., Wood, C.M., and Walsh, P.J. 2013. Transcriptome responses in the rectal gland of fed and fasted spiny dogfish shark (Squalus acanthias) determined by suppression subtractive hybridization. *Comp. Biochem. Physiol. Part D Genomics Proteomics* **8**: 334–343.

Eöry, L. et al. 2015. Avianbase: a community resource for bird genomics. *Genome Biol.* **16**: 21.

Genomic Resources Development Consortium et al. 2015. Genomic Resources Notes Accepted 1 August 2014–30 September 2014. *Mol. Ecol. Resour.* **15**: 228–229.

Kersey, P.J. et al. 2016. Ensembl Genomes 2016: more genomes, more complexity. *Nucleic Acids Res.* **44**: D574–D580.

Leinonen, R., Sugawara, H., and Shumway, M. 2011. The Sequence Read Archive. *Nucleic Acids Res.* **39**: D19–D21.

Matsunami, M., Kitano, J., Kishida, O., Michimae, H., Miura, T., and Nishimura, K. 2015a. Data from: Transcriptome analysis of predator- and prey-induced phenotypic plasticity in the Hokkaido salamander (Hynobius retardatus). (Accessed July 28, 2016).

Matsunami, M., Kitano, J., Kishida, O., Michimae, H., Miura, T., and Nishimura, K. 2015b. Transcriptome analysis of predator- and prey-induced phenotypic plasticity in the Hokkaido salamander (Hynobius retardatus). *Mol. Ecol.* **24**: 3064–3076.

Nourisson, C., Carneiro, M., Vallinoto, M., and Sequeira, F. 2014. Data from: “De novo transcriptome assembly and polymorphism detection in ecological important widely distributed Neotropical toads from the Rhinella marina species complex (Anura: Bufonidade)” in Genomic Resources Notes Accepted 1 August 2014-30 September 2014. (Accessed July 28, 2016).

Qiao, L., Yang, W., Fu, J., and Song, Z. 2013. Transcriptome Profile of the Green Odorous Frog (Odorrana margaretae). *PLoS ONE* **8**: e75211.

Richards, V.P., Suzuki, H., Stanhope, M.J., and Shivji, M.S. 2013. Characterization of the heart transcriptome of the white shark (Carcharodon carcharias). *BMC Genomics* **14**: 697.

Robertson, L.S., and Cornman, R.S. 2013. Data from: Transcriptome resources for the frogs Lithobates clamitans and Pseudacris regilla, emphasizing antimicrobial peptides and conserved loci for phylogenetics. (Accessed July 28, 2016).

Robertson, L.S., and Cornman, R.S. 2014. Transcriptome resources for the frogs Lithobates clamitans and Pseudacris regilla, emphasizing antimicrobial peptides and conserved loci for phylogenetics. *Mol. Ecol. Resour.* **14**: 178–183.

Tzika, A.C., Ullate-Agote, A., Grbic, D., and Milinkovitch, M.C. 2015. Reptilian Transcriptomes v2.0:

An Extensive Resource for Sauropsida Genomics and Transcriptomics. *Genome Biol. Evol.* **7**: 1827–1841.

Wang, K., Omotezako, T., Kishi, K., Nishida, H., and Onuma, T.A. 2015. Maternal and zygotic transcriptomes in the appendicularian, Oikopleura dioica: novel protein-encoding genes, intra-species sequence variations, and trans-spliced RNA leader. *Dev. Genes Evol.* **225**: 149–159.

Wang, Q. et al. 2012. Community annotation and bioinformatics workforce development in concert—Little Skate Genome Annotation Workshops and Jamborees. *Database J. Biol. Databases Curation* **2012**. http://www.ncbi.nlm.nih.gov/pmc/articles/PMC3308154/ (Accessed July 28, 2016).

Wielstra, B., Duijm, E., Lagler, P., Lammers, Y., Meilink, W.R.M., Ziermann, J.M., and Arntzen, J.W. 2014. Data from: Parallel tagged amplicon sequencing of transcriptome-based genetic markers for Triturus newts with the Ion Torrent next-generation sequencing platform. (Accessed July 28, 2016).

Wielstra, B., Duijm, E., Lagler, P., Lammers, Y., Meilink, W.R.M., Ziermann, J.M., and Arntzen, J.W. 2014. Parallel tagged amplicon sequencing of transcriptome-based genetic markers for Triturus newts with the Ion Torrent next-generation sequencing platform. *Mol. Ecol. Resour.* **14**: 1080–1089.
